# Global Sensitivity Analysis of Four Chamber Heart Hemodynamics Using Surrogate Models

**DOI:** 10.1109/TBME.2022.3163428

**Published:** 2022-03-30

**Authors:** Elias Karabelas, Stefano Longobardi, Jana Fuchsberger, Orod Razeghi, Cristobal Rodero, Marina Strocchi, Ronak Rajani, Gundolf Haase, Gernot Plank, Steven Niederer

**Affiliations:** Institute of Mathematics and Scientific ComputingUniversity of Graz 27267 Austria; Cardiac Electromechanics Research Group, School of Biomedical Engineering and Imaging SciencesKing’s College London 4616 U.K.; Research IT Services DepartmentUniversity College London 4919 U.K.; Department of Adult EchocardiographyGuy’s and St Thomas’ Hospitals NHS Foundation Trust 369752 U.K.; Gottfried Schatz Research Center (for Cell Signaling, Metabolism and Aging), Division BiophysicsMedical University of Graz 31475 Austria; Cardiac Electromechanics Research Group, School of Biomedical Engineering and Imaging SciencesKing’s College London 4616 SE1 7EH London U.K.

**Keywords:** Biomedical computing, finite element analysis, fluid dynamics, gaussian processes, scientific computing

## Abstract

Computational Fluid Dynamics (CFD) is used to assist in designing artificial valves and planning procedures, focusing on local flow features. However, assessing the impact on overall cardiovascular function or predicting longer-term outcomes may requires more comprehensive whole heart CFD models. Fitting such models to patient data requires numerous computationally expensive simulations, and depends on specific clinical measurements to constrain model parameters, hampering clinical adoption. Surrogate models can help to accelerate the fitting process while accounting for the added uncertainty. We create a validated patient-specific four-chamber heart CFD model based on the Navier-Stokes-Brinkman (NSB) equations and test Gaussian Process Emulators (GPEs) as a surrogate model for performing a variance-based global sensitivity analysis (GSA). GSA identified preload as the dominant driver of flow in both the right and left side of the heart, respectively. Left-right differences were seen in terms of vascular outflow resistances, with pulmonary artery resistance having a much larger impact on flow than aortic resistance. Our results suggest that GPEs can be used to identify parameters in personalized whole heart CFD models, and highlight the importance of accurate preload measurements.

## I. Introduction

Valvular heart disease is a growing problem with limited pharmacological therapies [1]. Patients with valvular malfunctions are at high risk of developing cardiovascular diseases (CVD) [2]. Valve treatments rely on invasive surgery or catheter-based implanted valves [3]. Choosing the best option for each patient remains a challenge [4].

However, our understanding of how valvular diseases affect the heart and cardiovascular system as a whole remains incomplete. Mechanistic models [5] encapsulate our knowledge of physiology and the underlying fundamental laws of physics. They provide a framework to integrate experimental and clinical data, enabling the identification of mechanisms and/or the prediction of outcomes, even under unseen scenarios without the need for retraining [6]. Computational fluid dynamics (CFD) is routinely used for designing valves [7] and guiding implantation planning [8]. These simulations focus on modeling local blood flow across the valve and do not consider blood flow in the wider heart. Simulating blood flow in the whole heart can be important when estimating pressure gradients in the left ventricular outflow tract in transcatheter mitral valve implants (TMVI) [9], or when considering ventricle size in transcatheter aortic valve implants (TAVI) [10]. However, patient-specfic simulations of blood flow in the whole heart requires parameters and boundary conditions to be tuned to an individual, requiring numerous expensive simulations. There is a need to reduce the computational cost of simulations and to focus simulations on tuning important parameters. Previous studies have performed local sensitivity analysis in simplified models, see for example [11], [12], however, these fail to provide an estimate of global and multi-factorial sensitivity. Identifying the key parameters that need to be personalized will both focus clinical measurements of key patient phenotypes and reduce the parameter space that needs to be explored to personalize the models.

The gold standard for modeling valves casts blood-valve interaction as a transient fluid-structure interaction (FSI) problem. Recent advances [13]–[15] show the potential of fully coupled FSI models. However, computational costs and patient-specific parametrization [16] still pose major obstacles, hindering a swift clinical translation. Immersed boundary methods (IBM) [17] have proven to be a promising alternative, combining computational efficiency, ease of implementation, and numerical stability [18], especially when applied to heart valve modeling [19]–[21].

In this study we create and validate a patient-specific model of blood flow across the four chambers of the heart using and extending the residual-based variational multiscale formulation (RBVMS) [22] of the arbitrary Lagrangian-Eulerian Navier-Stokes-Brinkman equations (ALE-NSB) [23]–[26]. We test the ability of machine learning-based GPEs, which approximate the model and estimate the uncertainty in the approximation, to provide a low-cost surrogate for the full physics-based model. While surrogate modeling of certain cardiac function models has gathered some traction in the context of physics-informed neural networks (PINNs) [27]–[29] this work is, to the best of the authors knowledge, the first attempt at developing a GPE based surrogate model in the context of four-chamber hemodynamics. As such it lays ground work for future studies including Bayesian history matching and inverse problems for inferring key hemodynamic biomarkers (such as atrial preload) in a non-invasive way. As added benefit, GPEs are designed to deal with model uncertainties [30] which are common issues in clincial practice. Using GPEs, we perform a variance-based GSA over parameters governing flow in the left and right heart to determine which of those are most important and need to be accurately personalized for patient-specific predictions.

## II. Methods

### A. Ethics Declaration

This study uses a fully anonymized data set collected at Guy’s and St Thomas’ Hospital, London, United Kingdom, as part of standard of care.

### B. Data Acquisition

The patient received a ECG-gated cardiac CT angiography. Clinically indicated MDCT was performed as the standard of care using the hospital’s 3$^\text{rd}$ generation dual-source CT system (SOMATOM Force, Siemens Healthcare, Forchheim, Germany) equipped with an integrated high-resolution detector (Stellar Technology, Siemens). Intravenous contrast (Omnipaque, GE Healthcare, Princeton, NJ) was administered using power injector ($5$$\mathrm{m}$$\mathrm{L}$$\mathrm{/}$$\mathrm{s}$) via the ante-cubital vein followed by saline flush (60–90 $\mathrm{m}\mathrm{L}$ total contrast volume). Descending aorta contrast-triggered (100 Hounsfield units [HU] at 120kVp), electrocardiogram (ECG)-gated formal CT data acquisition was begun on reaching this threshold with a 10 s delay. CT parameters include a slice collimation of $192$×$0.6$ $\mathrm{m}\mathrm{m}$, gantry rotation time of $250$ $\mathrm{m}$$\mathrm{s}$, pitch of $3.2$. Automated tube current modulation was performed using a reference tube current-time product of $400$ $\mathrm{m}$$\mathrm{A}$$\mathrm{s}$ and using automated attenuation-based tube voltage selection with a reference tube potential of 120 kVp. Initial retrospective ECG-gated scans were reconstructed in $5{\%}$ phase increments throughout the cardiac cycle using iterative reconstruction, slice thickness of $0.6$ $\mathrm{m}$$\mathrm{m}$ and an increment of $0.4$ $\mathrm{m}$$\mathrm{m}$. Patient data is summarized in Table I.

**TABLE I:** Patient Data

Parameter	Value
Left ventricular ejection fraction (LVEF)	$34{\%}$
Left ventricular end diastolic volume (LVEDV)	$414{\mathrm{m}\mathrm{L}}$
Left ventricular end systolic volume (LVESV)	$274{\mathrm{m}\mathrm{L}}$
Hear rate (HR)	83 bpm
Cardiac output (CO)	$11.62{\mathrm{L}\mathrm{/}\mathrm{min}}$
Systolic cuff pressure ($\text{P}_\text{sys}^\text{cuff}$)	$\text{97}\,\text{mmHg}$
Diastolic cuff pressure ($\text{P}_\text{dia}^\text{cuff}$)	$\text{57}~\text{mmHg}$
Gender	male
Age	$74$

### C. Model Generation

**Fig. 1.**
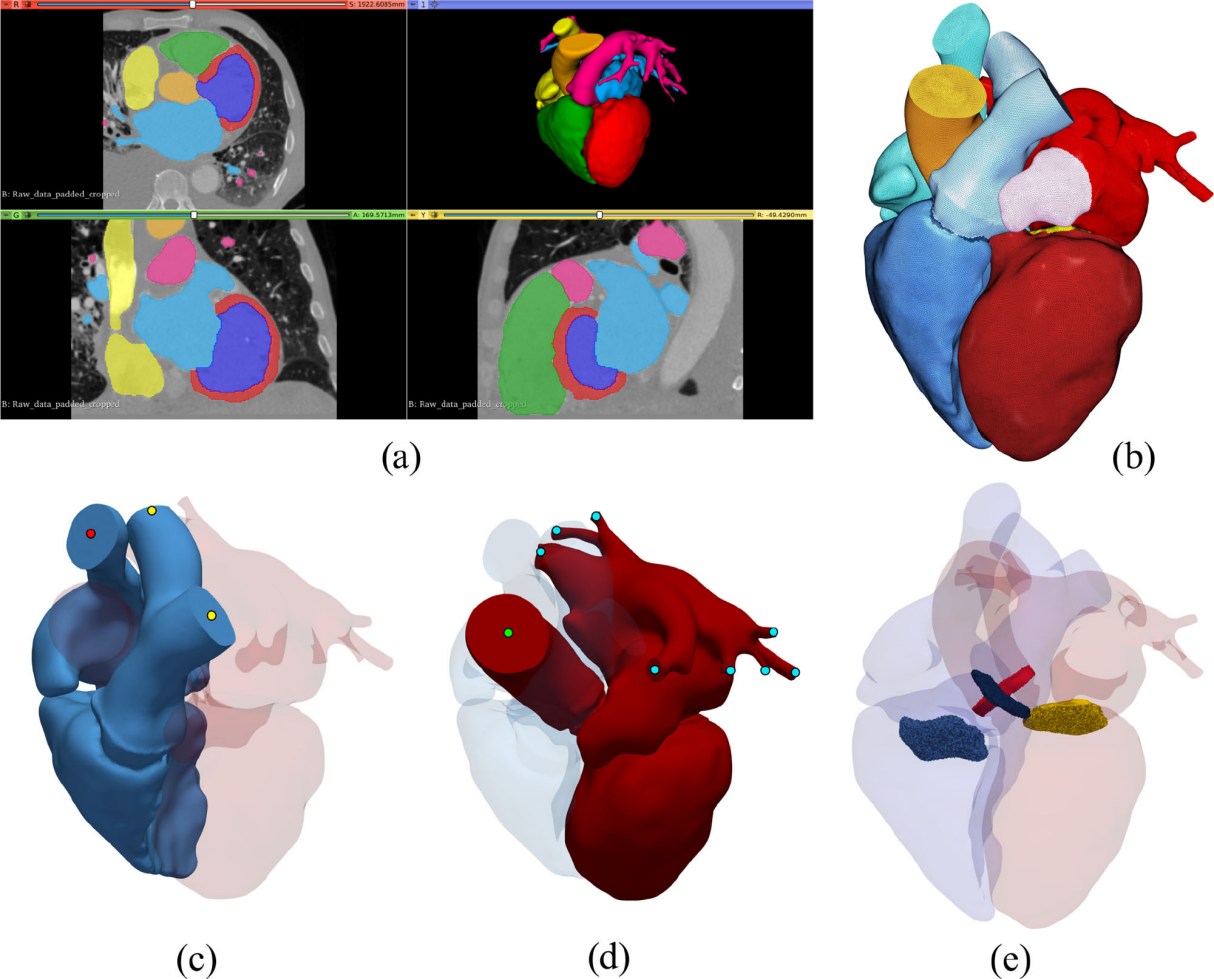
Whole heart model generation workflow. Shown are a) the pre-final segmentation in Slicer before automatically adding valve regions, b) the final multi-label mesh, the outflow boundaries for c) the right heart, marked by red and yellow circles, and d) the left heart, marked by green and blue circles, and e) the automatically generated valve regions.

Cardiac anatomy was automatically segmented from the CT DICOM images [31]–[33], to provide labels for all cardiac chambers and major vessels [Fig. 1(a)]. Additional post processing was performed using Seg3D and Slicer to obtain 16 labels comprising left ventricle (LV), right ventricle (RV), left atrium (LA), right atrium (RA), aorta (AO), and pulmonary artery (PA) blood pools as well as labels encoding the locations of aortic valve (AV), mitral valve (MV), pulmonary valve (PV) and tricuspid valve (TV). Valve labels were automatically generated as thin voxel regions between compartment regions see Fig. 1(e). Multilabel segmentations were used to create an unstructured finite element surface mesh using CGAL, which served as input for the unstructured volumetric mesh generation, including three prismatic boundary layers, using the software package meshtool [34] [see Fig. 1(b)]. Cardiac kinematics was extracted over one cardiac cycle by non-rigid registration, using the sparse free-form deformation (SFFD) technique [35] that extends the classic FFD approach and recovers smoother displacement fields [36], [37].

### D. Computational Methods & Simulation

**Fig. 2.**
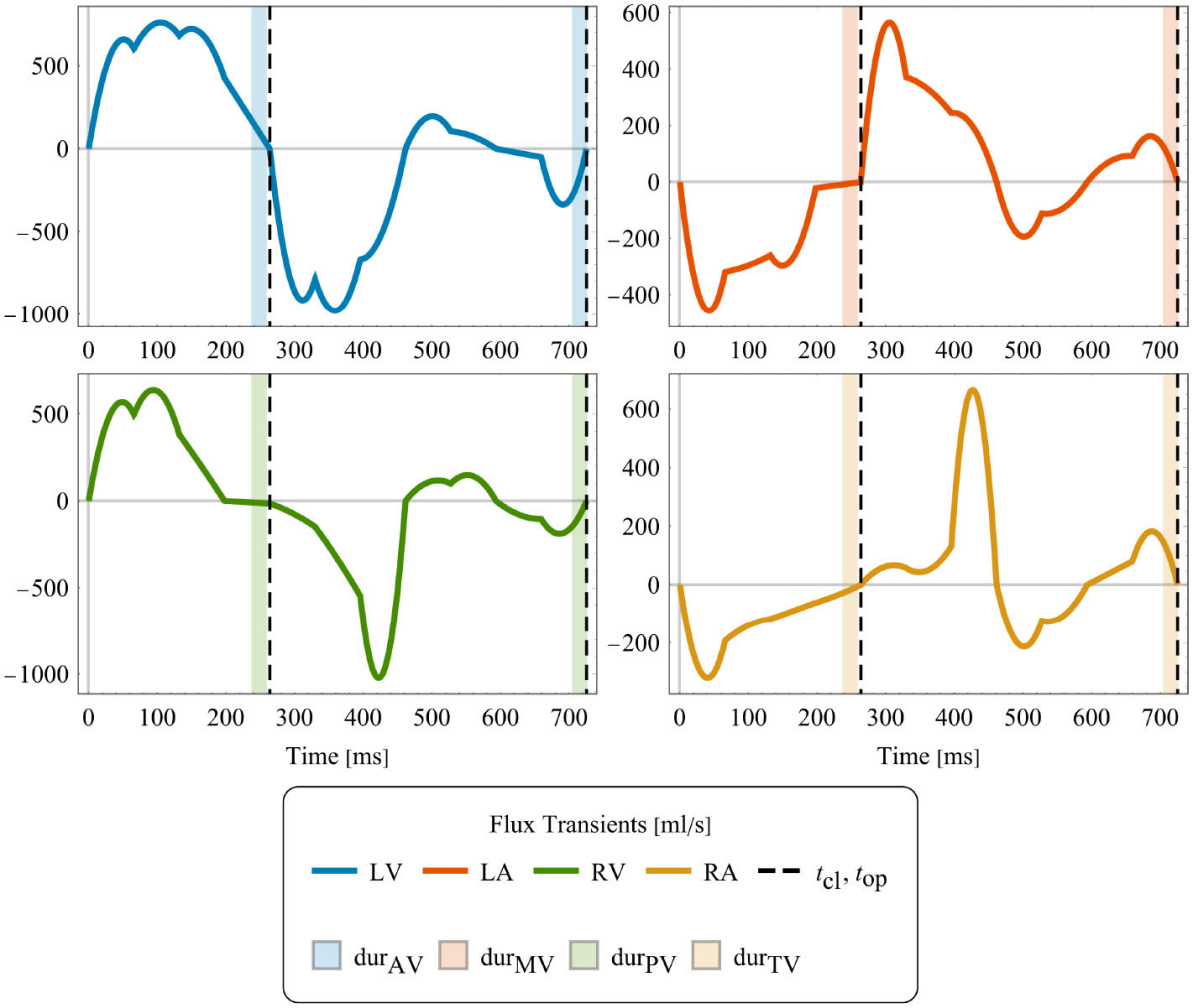
Fluxes computed from volume transients of Fig. 3. Dashed lines indicated timings of valves switching, with opaque bars indicating the duration of switching.

**Fig. 3.**
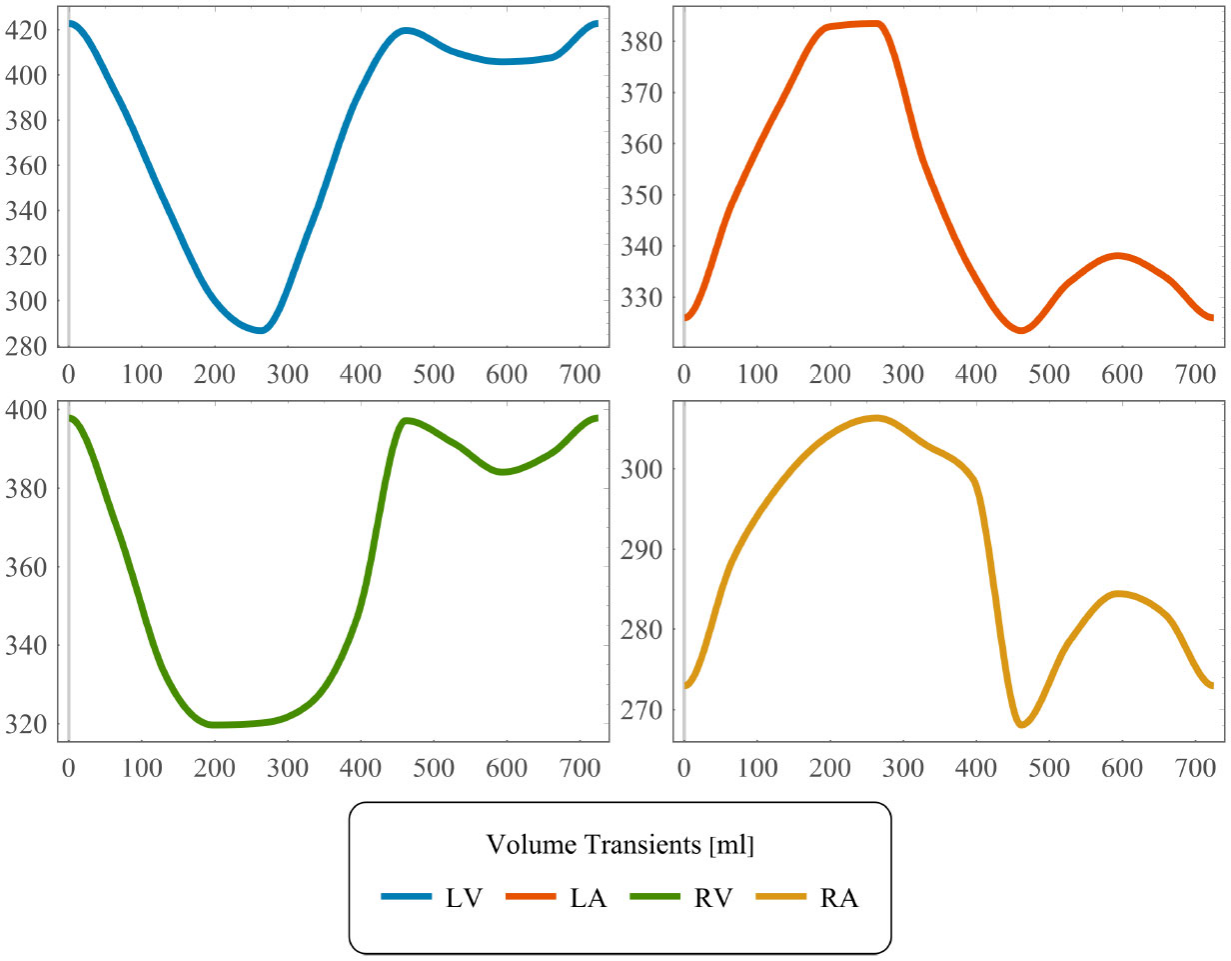
Volume transients extracted from the registered mesh motion of LV, RV, LA and RA blood pool.

Image derived kinematics was used as input to drive the CFD model of whole-heart hemodynamics. With prescribed motion, blood flow in the left and right heart can be simulated independently. Assuming Newtonian blood flow, hemodynamics is modeled with an arbitrary Lagrangian-Eulerian (ALE) formulation of the Navier-Stokes equations [38], [39]. The effect of heart valves upon blood flow is taken into account by including an ad-hoc extension to the ALE-Navier-Stokes-Brinkman (ALE-NSB) equations with an added Darcy drag term penalizing flow in the areas covered by the valves [23], [24], [40]. Extensions required for moving domains are explained in more detail in Supplement S.I. Computational domains labeled as valves are parameterized by a penalty parameter $\kappa _*$, modeling vanishing permeability, with $*$ denoting any of the four heart valves, AV, MV, PV, TV, and the duration $\text{dur}_*$ (see Fig. 2 for an illustration) of valve opening and closing. A RBVMS discretization is used [22], adapted to the ALE-NSB equations. A generalized-$\alpha$ integrator [41] with $\rho _\infty = 0.2$ is employed for time discretization and the arising non-linear systems are solved with an inexact Newton-Raphson method [42]. Mesh convergence was investigated using Pope’s criterion [43], see Supplement S.IV. Domain motion was extended into the interior of the blood pool using a linear elastic model optimized for retaining finite element quality. Dirichlet displacement boundary conditions are used at the blood pool walls enforcing a velocity matching the time derivative of the registered cardiac motion. On the arterial outlets (aorta and pulmonary artery) we used $0D$ three element Windkessel models [44]. Windkessel parameters of systemic circulation comprising characteristic impedance, $\mathrm{Z}_\text{WK}$, resistance $\mathrm{R}_\text{WK}$ and compliance $\mathrm{C}_\text{WK}$ were determined from cuff pressure measurements [45], [46]. This resulted in $\mathrm{R}_\text{WK}=49.89\,{\mathrm{k}\mathrm{Pa}\mathrm{m}\mathrm{s}\mathrm{/}\mathrm{m}\mathrm{L}}$. Values for $\mathrm{Z}_\text{WK}$ and $\mathrm{C}_\text{WK}$ were determined as $0.05 \mathrm{R}_\text{WK}$ and $\mathrm{C}_\text{WK}=\frac{\text{HR}}{\mathrm{R}_\text{WK}}$ respectively. As no pressure measurements were available for the right heart, Windkessel parameters for the pulmonary circulation were estimated by assuming a default value of $\text{14}\,\text{mmHg}$ for mean pulmonary artery pressure [47] and estimating Windkessel parameters from this value. RV cardiac output was estimated from its end diastolic and end systolic volume, with the latter estimated from the volume transients in Fig. 3. At the other outlets pressures $p_\text{LA}=10\,\text{mmHg}$ and $p_\text{RA}=5\,\text{mmHg}$ were prescribed. The location of all outlets are illustrated in Fig. 1(c) and (d). For numerical stability the directional do-nothing outflow stabilization [48] was used.

### E. Global Sensitivity Analysis

To quantify the impact of input parameters on the total variance of output features global sensitivity analysis (GSA) using *Gaussian process emulation* (GPE) was employed to replace the highly non-linear computationally expensive map from parameters to features with a fast-evaluating, probabilistic surrogate map. We selected $D$ parameters and $M$ characteristic output features for the studied model. GPEs were trained following [49]. Briefly, we used a $\approx 10D$ sized sample drawn from a Latin hypercube design in the $D$-dimensional parameter space with initial ranges given by $\pm 25{\%}$ perturbation around the baseline values. Model simulations were carried out for each of these parameter sets and the successfully completed simulations were collected to build the training dataset. Simulations where CFD simulation failed to converge were discarded. GPEs were defined as the sum of a deterministic mean function and a stochastic process [50] while the stochastic process is a centered zero-mean Gaussian process with stationary Matérn covariance function [51]. The model likelihood was taken to be Gaussian, i.e. the learning sample observations are modeled to be affected by an additive, independent and identically distributed noise.

### F. Computational Framework

#### 1) Computational Fluid Dynamics

The discretized and linearized block system of the ALE-NSB equations was solved for each Newton-Raphson iteration and every time step. A flexible generalized minimal residual method (fGMRES) and efficient preconditioning based on the libraries PETSc and hypre/BoomerAMG were employed. CFD model and calculation of residence times have been implemented in an extension of the Cardiac Arrhythmia Research Package (carpentry) [52]. Parallel performance and scalability of carpentry has been previously investigated in [45], [53]. Details on numerical aspects are provided in Supplement S.I.C.

#### 2) GPE Training

All the GPE’s (hyper)parameters were jointly optimized by minimization of the negative model log-marginal likelihood [30] using GPErks emulation tool based on the GPyTorch Python library which itself uses the ADAM optimizer [54]. Univariate GPEs were trained to predict each output feature using a $5$-fold cross-validation process. Results are given in Supplement S.VI. GPEs’ accuracy was evaluated using the average $R^{2}$-score across the obtained scores when testing the emulators on the respective left-out parts of the dataset. The so trained GPEs were used as emulators for the global sensitivity analysis. Model outputs’ sensitivity to parameters was characterized by Sobol’ first-order $S_{1}$ and total effects $S_{T}$ [55].

### G. Data Analysis

Pressure gradients and differences as well as flow velocities were calculated by computing spatial averages over spherical regions chosen as observation sites, see Fig. 5. All chosen regions did not intersect the prismatic boundary layers. Output features used for training were calculated from derived quantities by temporal averaging, or taking the temporal maximum over the whole cardiac cycle.

**Fig. 4.**
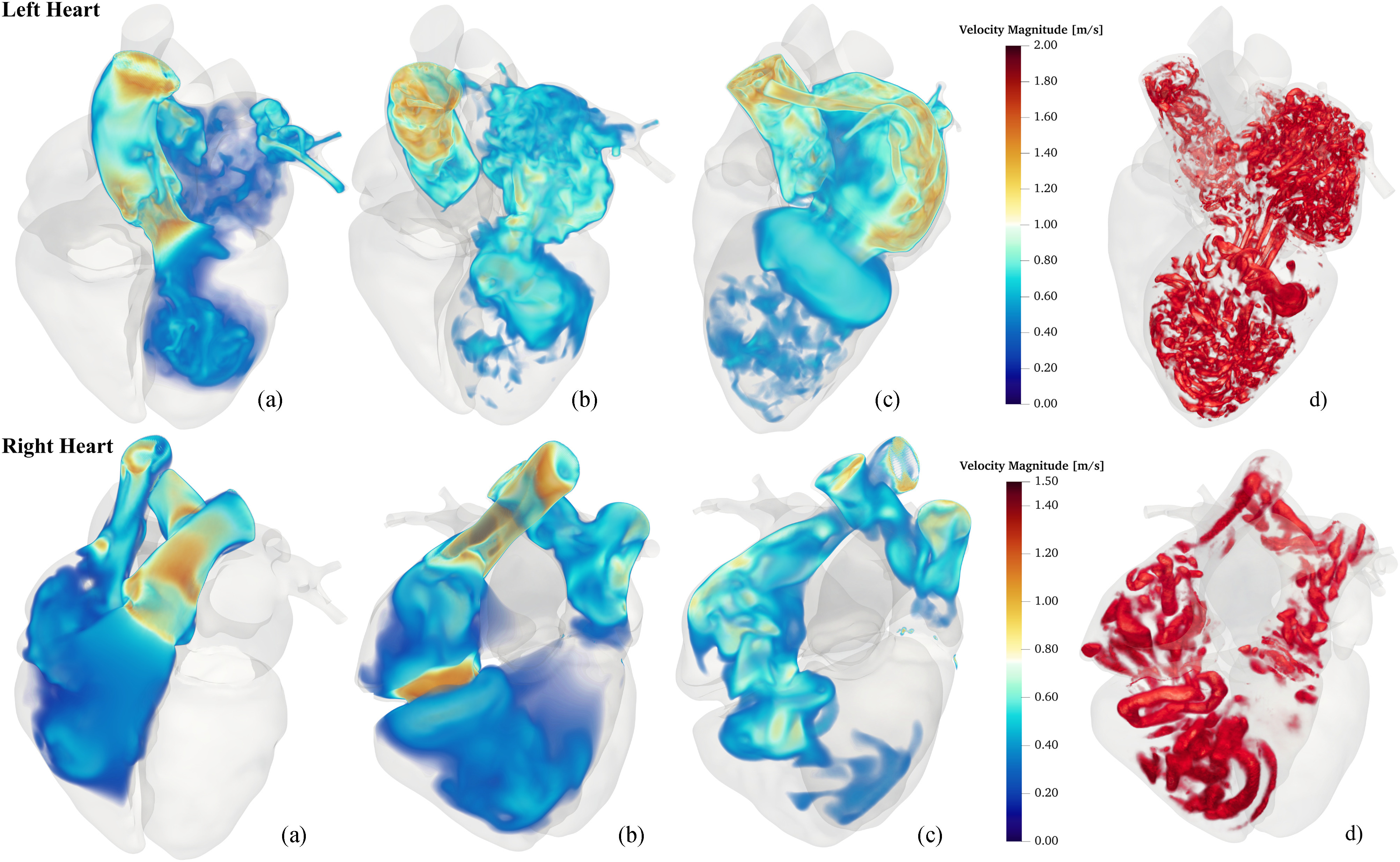
CFD results show left (top panels) and right (bottom panels) velocity magnitude of heart blood flow at a) peak systole, b) end of systole, and c) peak diastole, and d) isosurfaces of the strain normalized $Q$ criterion at peak diastole for threshold value 2.5.

**Fig. 5.**
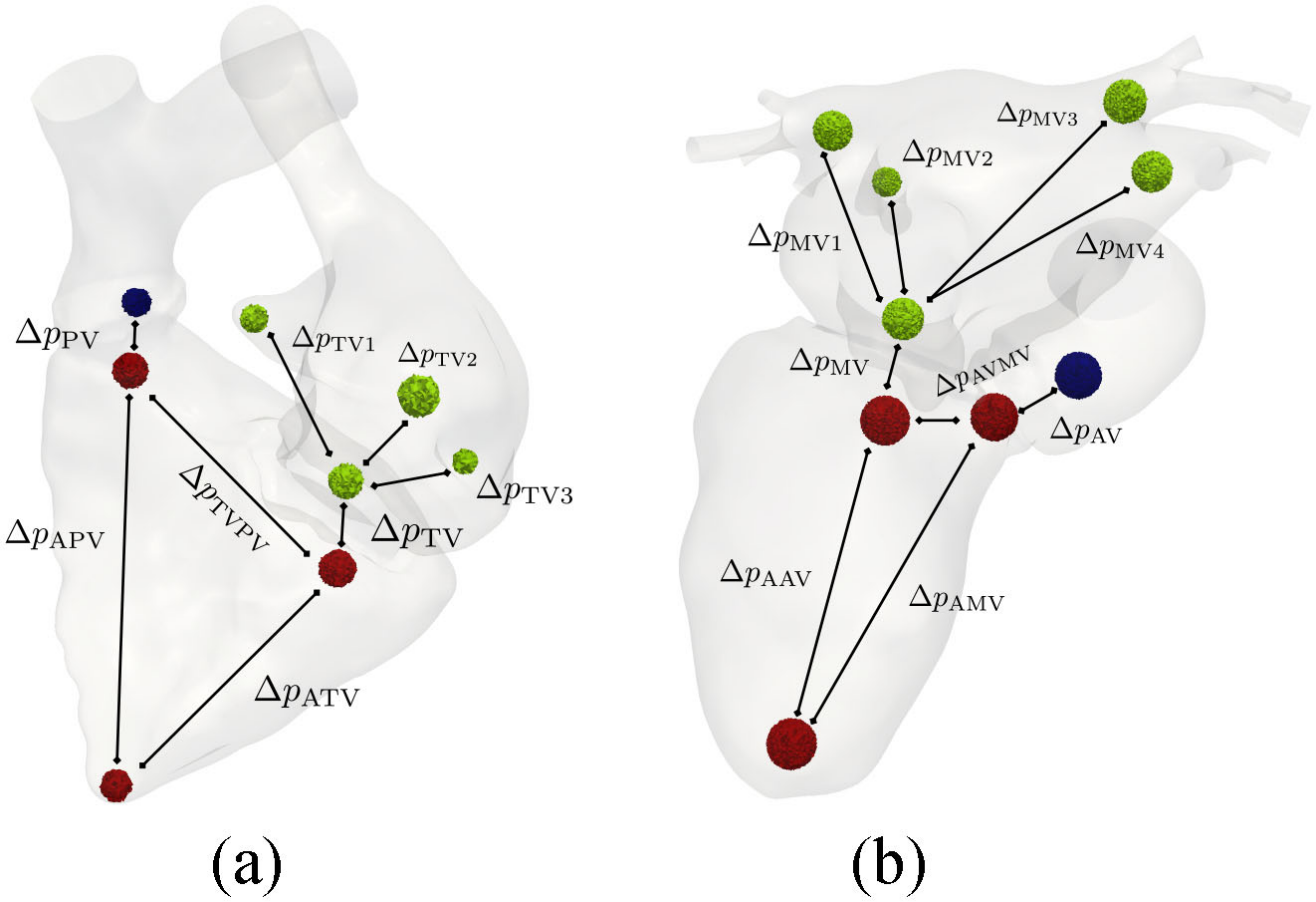
Illustration of areas in the left and right heart used to compute pressure drops and differences respectively. A black line denotes that the pressure difference between those areas is calculated.

## III. Results

### A. Simulation

Four heart beats were simulated at a time step of $\Delta t = 0.3625\,{\mathrm{m}\mathrm{s}}$ resulting in 16000 time steps. Simulations were carried out on the *Vienna Scientific Cluster 4* (VSC4) using 1152 MPI processes and 672 MPI processes, with an average run time per time step of $18{\mathrm{s}}$ and $9{\mathrm{s}}$ and a total run time of $80{\mathrm{h}}$ and $40{\mathrm{h}}$ for left and right heart simulations, respectively. Volume renderings of the velocity magnitude at various time instances are shown in Fig. 4. The large-scale flow characteristics in both ventricles is the formation of an asymmetric vortex ring [Fig. 4(c) and (e)] next to the MV and TV traveling towards the apex, also apparent in the visualization of the strain-normalized $Q$ criterion in Fig. 4(d) and (h). As expected, jet formation is witnessed at the opening of the heart valves, see rightmost subfigures of Fig. 4. Furthermore, flow in the AO shows strong non-laminar behavior and increased flow speeds can be observed in the upper areas of the LA. A video showing the final heart beat is available as supplement.

### B. Global Sensitivity Analysis Using Surrogate Models

We performed a GSA for both sides of the heart as outlined in Section II-E. First, we used $D=6$ parameters (summarized in Table II) as key regulators of our left heart model, and we characterized the model behavior at a specific set of parameters using $M=16$ features with notation and baseline values summarized in Table III. More specific, we used the following output features: mean systolic pressure gradient over aortic valve (AV), $\Delta \mathrm{p}_\text{AV}$, and mean diastolic pressure gradient over mitral valve (MV), $\Delta \mathrm{p}_\text{MV}$, as defined in [56]; mean pressure difference between four landmark points in the LA and MV, $\Delta \mathrm{p}_{\text{MV}1,2,3,4}$; mean pressure difference between apex and MV, $\Delta \mathrm{p}_{\text{AMV}}$; mean pressure difference between apex and AV, $\Delta \mathrm{p}_{\text{AAV}}$; mean pressure difference between AV and MV, $\Delta \mathrm{p}_{\text{AVMV}}$; mean pressure gradient over PV, $\Delta \mathrm{p}_{\text{PV}}$; mean pressure gradient over TV, $\Delta \mathrm{p}_{\text{TV}}$; mean pressure difference between four landmark points in the RA and TV, $\Delta \mathrm{p}_{\text{TV}1,2,3}$; mean kinetic energy LV, $\mathrm{E}_{\mathrm{k},\text{LV}}$; mean kinetic energy AO, $\mathrm{E}_{\mathrm{k},\text{AO}}$; mean kinetic energy LA, $\mathrm{E}_{\mathrm{k},\text{LA}}$; mean kinetic energy RV, $\mathrm{E}_{\mathrm{k},\text{RV}}$; mean kinetic energy PV, $\mathrm{E}_{\mathrm{k},\text{PV}}$; mean kinetic energy RA, $\mathrm{E}_{\mathrm{k},\text{RA}}$; average residence time, LV $\text{RT}_\text{LV}$; average residence time, RV $\text{RT}_\text{RV}$; average residence time, left atrial appendage (LAAPP) $\text{RT}_\text{APP}$; maximal velocity magnitude over AV, $\text{maxv}_{\text{AV}}$, MV, $\text{maxv}_{\text{MV}}$, PV, $\text{maxv}_{\text{PV}}$, and TV, $\text{maxv}_{\text{TV}}$. Residence times were calculated using an continuum approach described in [57] solved with a novel flux corrected transport finite element method (FCT-FEM) inspired from [58] adapted to moving grids. Details are given in the Supplement S.III.

**TABLE II:** Parameters Identified for GPE Training

Parameter	Range	Description
$R_{\text{WK},\text{AO}}$	37.46 $\frac{\text{kPa ms}}{\text{mL}}$ to 62.32$\frac{\text{kPa ms}}{\text{mL}}$	AO Windkessel resistance
$R_{\text{WK},\text{PA}}$	27.81 $\frac{\text{kPa ms}}{\text{mL}}$ to 46.21$\frac{\text{kPa ms}}{\text{mL}}$	PA Windkessel resistance
$p_\text{LA}$	7.5 mmHg to 12.5 mmHg	LA outlet pressure
$p_\text{RA}$	3.5 mmHg to 8.5 mmHg	RA outlet pressure
$\kappa _\text{AV}$	$1 \times 10^{-5\,\,\mathrm{to}\,\,1e-9}$	AV penalization parameter
$\kappa _\text{MV}$	$1 \times 10^{-5\,\,\mathrm{to}\,\,1e-9}$	MV penalization parameter
$\text{dur}_{\mathrm{*}}$	$11.25 \,\mathrm{m}\mathrm{s}\,\mathrm{to}\, 18.75 \,\mathrm{m}\mathrm{s}$	Valve transition times with $* \in \lbrace \text{AV}, \text{MV}, \text{PV}, \text{TV}\rbrace$

**TABLE III:** Output Features for GPE Training With Reference Values Extracted From CFD Simulations. Reported are Temporal Means, Except for Velocities Reported as Temporal Maxima. Clinical Measurements If Reported are Given as Means of Three Measurements

Parameter	*in silico* Reference Value	Clinical Measurements
$\Delta \mathrm{p}_\text{AV}$	$4.61$ mmHg	$5.0$ mmHg
$\Delta \mathrm{p}_\text{MV}$	$2.71$ mmHg	$2.38$ mmHg
$\Delta \mathrm{p}_{\text{MV}1,2,3,4}$	$$\matrix{-0.0106\cr 0.225 \cr 0.183 \cr -0.0025}\quad \hbox{mmHg}$$	
$\Delta \mathrm{p}_{\text{AMV}}$	$1.732$ mmHg	
$\Delta \mathrm{p}_{\text{AAV}}$	$1.60$ mmHg	
$\Delta \mathrm{p}_{\text{AVMV}}$	$0.21$ mmHg	
$\Delta \mathrm{p}_{\text{PV}}$	$2.35$ mmHg	$3.0$ mmHg
$\Delta \mathrm{p}_{\text{TV}}$	$5.73$ mmHg	$47.0$ mmHg
$\Delta \mathrm{p}_{\text{TV}1,2,3}$	$$\matrix{0.204\cr 0.342 \cr 0.228}\quad \hbox{mmHg}$$	
$\mathrm{E}_{\mathrm{k},\text{LV}}$	$16.71$ $\mathrm{m}$ $\mathrm{J}$	
$\mathrm{E}_{\mathrm{k},\text{AO}}$	$22.59$ $\mathrm{m}$ $\mathrm{J}$	
$\mathrm{E}_{\mathrm{k},\text{LA}}$	$23.33$ $\mathrm{m}$ $\mathrm{J}$	
$\mathrm{E}_{\mathrm{k},\text{RV}}$	$3.65$ $\mathrm{m}$ $\mathrm{J}$	
$\mathrm{E}_{\mathrm{k},\text{PV}}$	$5.51$ $\mathrm{m}$ $\mathrm{J}$	
$\mathrm{E}_{\mathrm{k},\text{RA}}$	$9.59$ $\mathrm{m}$ $\mathrm{J}$	
$\text{RT}_\text{LV}$	$0.811$ $\mathrm{s}$	
$\text{RT}_\text{APP}$	$0.854$ $\mathrm{s}$	
$\text{RT}_\text{RV}$	$0.91$ $\mathrm{s}$	
$\text{maxv}_{\text{AV}}$	$1.13$ $\mathrm{m}$ $\mathrm{/}$ $\mathrm{s}$	$1.15$ $\mathrm{m}$ $\mathrm{/}$ $\mathrm{s}$
$\text{maxv}_{\text{MV}}$	$0.73$ $\mathrm{m}$ $\mathrm{/}$ $\mathrm{s}$	$0.81$ $\mathrm{m}$ $\mathrm{/}$ $\mathrm{s}$
$\text{maxv}_{\text{PV}}$	$0.71$ $\mathrm{m}$ $\mathrm{/}$ $\mathrm{s}$	$0.814$ $\mathrm{m}$ $\mathrm{/}$ $\mathrm{s}$
$\text{maxv}_{\text{TV}}$	$0.57$ $\mathrm{m}$ $\mathrm{/}$ $\mathrm{s}$	$3.43$ $\mathrm{m}$ $\mathrm{/}$ $\mathrm{s}$

**Fig. 6.**
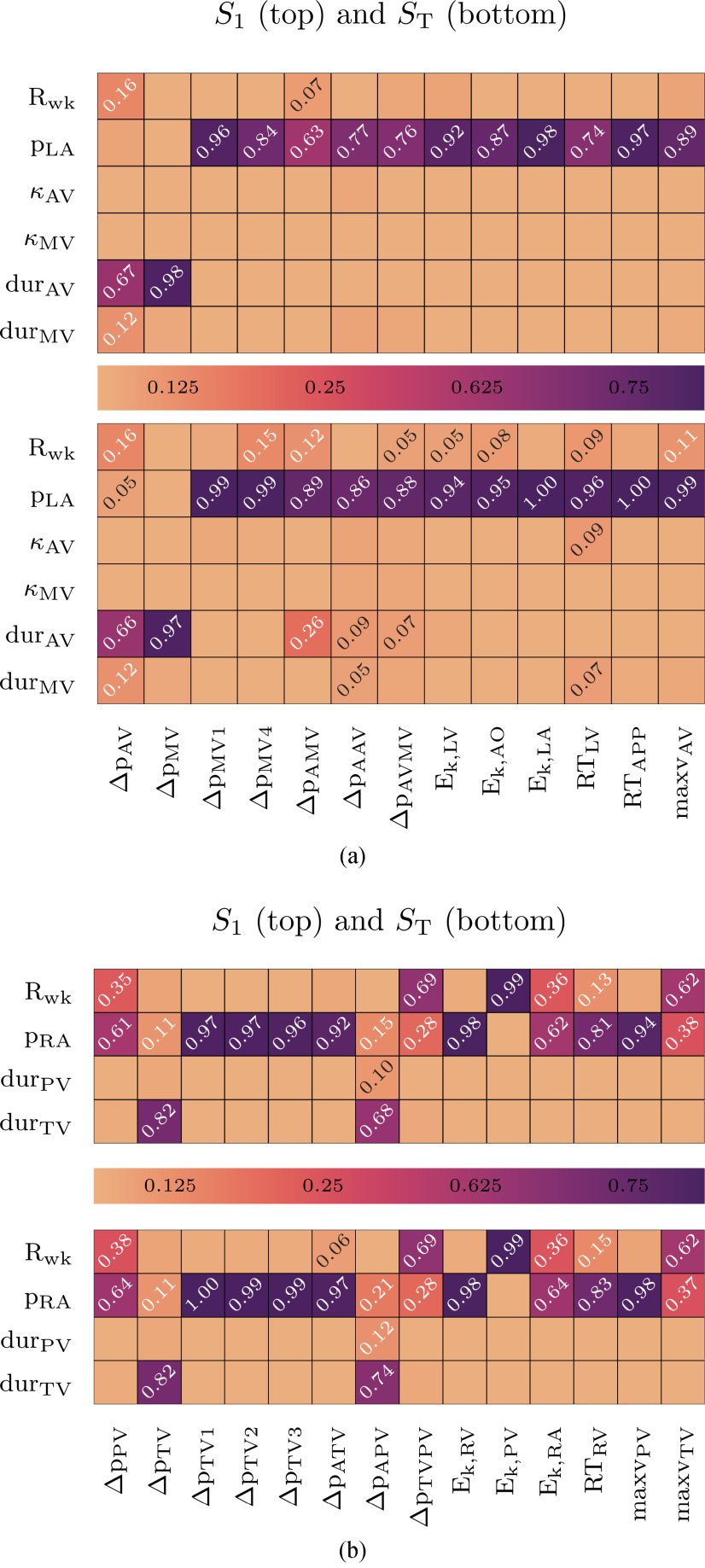
Heat maps of first and total order Sobol indices for the a) left heart and b) right heart GSA.

As described in Section II-E, we used GPErks to incorporate full GPE’s posterior distribution samples to estimate the first and total Sobol’ indices $S_{1}$ and $S_{T}$ using Saltelli’s method [59] with $n=10000$ samples drawn from each GPE. Sobol indices were calculated with the help of SALib python library [60]. Only GPEs having a mean $R^{2}$ test score $>0.5$ were used for indices calculation. This resulted in excluding features $\text{maxv}_\text{MV}$, $\Delta p_\text{MV2}$, and $\Delta p_\text{MV3}$ from the analysis. Parameters with resulting indices below $0.01$ were considered to have no/negligible effect. The resulting indices are summarized as heat-maps in Fig. 6(a). From GSA we concluded that the penalization parameters $\kappa _\text{AV}$, and $\kappa _\text{MV}$ have no or negligible effect and feature $p_\text{LA}$ has a strong effect. The same procedure was carried out for the right bloodpool model with penalization parameters $\kappa _*$ removed from the training phase due to negligible influence. We chose similar output features summarized in Table III. Results are summarized in Fig. 6(b) showing a strong effect of $p_\text{RA}$ and $R_\text{WK}$.

## IV. Discussion

Being able to identify key parameters and regulators in a hemodynamic CFD model of the human heart is paramount for personalization. However, personalization of four chamber CFD models is computationally expensive. Here we show that the use of ALE-NSB allows computationally tractable simulations, the GPE can be used to emulate most outputs, residence times can be calculated fast and precise using the novel algorithm in S.III, and that pre load is the key parameter in determining boundary driven four chamber heart CFD models. Our CFD simulations took between $10$$\mathrm{h}$ and $20$$\mathrm{h}$ per heart beat for the left or right side of the heart. This breaks down to an average wallclock time of $\approx$ $11$$\mathrm{s}$ for performing one nonlinear time step of the CFD simulator. Comparing our average wallclock times with other approaches, for instance $11$$\mathrm{s}$ reported in [61] using IBM, or $30$$\mathrm{s}$ - $50$$\mathrm{s}$ reported in [62] using a semi-implicit algorithm with higher order finite elements, or $50$$\mathrm{s}$ reported in [63] using a similar algorithm as in this manuscript, we find that our ALE-NSB method provides a competitive implementation putting us well into the forecasted optimal wallclock times for hemodynamic CFD simulations shown in [7].

Comparing with clinically measured data in Table III we saw good agreement for $\text{maxv}_{\mathrm{AV,MV,PV}}$ with relative error of $\approx$ $2$%, $10$%, and $13$% similarly for $\Delta p_{\mathrm{AV,MV,PV}}$ with relative error of $\approx$ $8$%, $13$%, and $24$%. Clinical data suggested a possible TV regurgitation. We did not aim to capture TV regurgitation in the simulations, and this likely explains the discrepancy in $\text{maxv}_\text{TV}$ and $\Delta p_\text{TV}$.

There is growing interest in using reduced order models and physics informed neural networks (PINNs) for accelerating or creating model surrogates [64]. Each method has its purpose, here we show that GPEs, which are both fast and provide an estimate of uncertainty can be used to emulate most, but not all, four chamber heart CFD simulation outputs using $\approx$ 10 simulations per parameter. To train our GPEs we used in total $180$ CFD simulations comprising 4 heart beats each. Executing those simulations took approximately $7700$$\mathrm{h}$ of wallclock time on the HPC clusters VSC4 (AT) and ARCHER (U.K.). Using those data sets we performed the first GSA of model parameters for informing cavity driven flow. Training of the GPEs and running GSA took approximately $5$$\mathrm{h}$. Running GSA without a surrogate model would have resulted in intractable amounts of CFD simulations highlighting the possible savings in computing time and resources.

Output features $\text{maxv}_\text{MV}$, $\Delta p_{\text{MV}2}$, and $\Delta p_{\text{MV}3}$ showed $R^{2}$ test scores below $0.5$. As the GPEs are based on nonlinear CFD simulations, it is hard to give a definite answer as to why those particular features were excluded. Possible explanations could be, underresolved CFD grids close to extraction points of the features, or lacking temporal resolution. We investigated whether there is any pattern in the failed samples which is not evident see Supplement S.VIII.

We identified the pre-load as a key variable in defining simulation clinical outputs, in both the atrial and ventricle flow in four chamber boundary driven flow simulations. This highlights the need to have an accurate estimate of pre-load when performing boundary driven CFD simulations. As blood flows from the atria to the ventricle and then out through the aorta (or pulmonary artery) the parameters that impact atrial flow will impact down stream flows. Conversely, the after-load properties only impact blood flow out of the ventricle and do not directly impact the atrial flow. This potentially explains the importance of pre-load over after-load in our simulations. Furthermore, we considered time averages over the complete heart beat. During systole, pressure signals are not sensitive to any of the input parameters. However, this changes in diastole and we provide an additional explanation in Supplement S.V. We used a CT based image derived wall motion to drive the CFD simulations. The wall motion is derived from retrospective gated CT that is acquired from only 3-4 heart beats. Over this short time frame there is no guarantee that the blood flow out of the right side of the heart precisely matches the inflow on the left side. Additionally, buffering effects of systemic compliances (i.e. lungs, and venes) can have influence on the mass fluxes recovered from CT. From our motion tracking we found that there is a 10 % difference in total blood volume in the left and right side of the heart. Concerning fluxes we found that over one simulated heartbeat we have 20 ml less inflow into the LA than outflow from the RV. On the other hand, there is 80 ml less inflow into the RA than outflow from the LV. This difference is likely explained by a degree of regurgitation that is not captured in our model. It is important to note that our findings are for the specific case of boundary driven flow and do not reflect the relative importance of pre-load and after-load in patients, where after-load can feedback on ventricle function, and hence atrial filling, so may play a far greater role physiologically. In this study we proposed a GPE as a low-cost emulator of large 3D CFD simulations. An alternate approach to using 3D CFD simulations with an GPE would have been to calibrate a 0D model to the patient data directly, or to 3D simulation results. 0D models have the benefit that they provide physics and physiology based constraints. These are more likely to work outside of the training data set. However, 0D models can only approximate a subset of 3D model outputs, do not capture 3D patient anatomy, cannot represent device interventions and do not provide an estimate of uncertainty. 3D and 0D models will have different use cases. We have shown how combining CFD and GPE allows global sensitivity analysis, that can readily be performed on 0D models, can also be applied to complex larger 3D models.

## V. Conclusion

In this work we presented full GSA based on GPE surrogate models for four chamber heart hemodynamics. We showed that modeling valves using a penalization approach is independent of numerical parameters. GSA revealed strong influences of left and right atrial pressure and medium influence of arterial and pulmonary arterial resistances. These results show the possibility and potential speedup using surrogate models to replace full-blown CFD models for human heart hemodynamics.

## Supplementary Materials

Supplementary materials
