# Supplementary material for: Global Sensitivity Analysis of Four Chamber Heart Hemodynamics Using Surrogate Models
Source: IEEE Trans Biomed Eng. 2022 Mar 30;69(10):3216–23. doi: 10.1109/TBME.2022.3163428 (PMC9491017; doi:10.1109/TBME.2022.3163428)
Supplement: Supplementary materials [file supp2-3163428.pdf]

Supplementary Material for "Global Sensitivity Analysis of Four Chamber Heart Hemodynamics Using Surrogate Models"

E. Karabelas, S. Longobardi, J. Fuchsberger, O. Razeghi, C. Rodero, R. Rajani, M. Strocchi, G. Haase, G. Plank, and S. Niederer

March 22, 2022

S.I. Residual-Based Variational Multiscale Formulation for Navier-Stokes-Brinkman Equations on moving domains

The Navier-Stokes-Brinkman (NSB) equations, originating from porous media theory, can be employed with the purpose of simulating viscous flow including complex shaped solid obstacles in a fluid domain, see [*S2*], and [*S1*, *S11*] for a in-depth mathematical analysis. The NSB model was successfully extended to moving obstacles and applied to model flapping insect flight in [*S13*]. In the present work, we use the NSB equations including the adaptation for moving obstacles as well as moving domains using the *arbitrary lagrangian Eulerian* (ALE) formulation [*S20*, *S22*, *S41*]:

$$
\begin{aligned}
&\rho\left(\frac{\partial}{\partial t}\boldsymbol{u} + (\boldsymbol{u}-\boldsymbol{w})\cdot\nabla\boldsymbol{u}\right) - \nabla\cdot\boldsymbol{\sigma}(\boldsymbol{u},p) + \frac{\mu}{K}(\boldsymbol{u}-\boldsymbol{u}_s) = 0 && \text{in } \mathbb{R}^+\times\Omega(t), && (1)\\
&\nabla\cdot\boldsymbol{u} = 0 && \text{in } \mathbb{R}^+\times\Omega(t), && (2)\\
&\boldsymbol{u} = \boldsymbol{w} && \text{on } \Gamma_{\text{noslip}}(t), && (3)\\
&\boldsymbol{\sigma}\boldsymbol{n} - \rho\beta((\boldsymbol{u}-\boldsymbol{w})\cdot\boldsymbol{n})_- = \boldsymbol{h} && \text{on } \Gamma_{\text{outflow}}(t), && (4)\\
&\boldsymbol{u} = \boldsymbol{g} && \text{on } \Gamma_{\text{inflow}}(t), && (5)\\
&\boldsymbol{u}|_{t=0} = \boldsymbol{u}_0, && && (6)
\end{aligned}
$$

with the time dependent fluid domain $\Omega(t)$ defined as

$$\Omega(t) := \{\, \boldsymbol{x} \mid \boldsymbol{x} = \boldsymbol{X} + \boldsymbol{d}(\boldsymbol{X},t), \forall \boldsymbol{X} \in \Omega_0 \,\},$$

using the ALE mapping $\boldsymbol{d}$ transforming an arbitrary reference configuration Ω_0 into the current fluid domain $\Omega(t)$. Here p, $\boldsymbol{u}$, and $\boldsymbol{w} := \frac{\partial}{\partial t}\boldsymbol{d}$ represent the fluid pressure, the flow velocity, and the ALE velocity respectively, μ is the dynamic viscosity and ρ the density. The volume penalization term $\frac{\mu}{K(t,\boldsymbol{x})}(\boldsymbol{u}(t,\boldsymbol{x}) - \boldsymbol{u}_s(t,\boldsymbol{x}))$ is commonly known as *Darcy drag* which is characterized by the permeability $K(t,\boldsymbol{x})$. Another interpretation of the *Darcy drag* can be given as resistance term, as for example aerodynamic drag is also interpreted as a resistance. In (1) the Darcy drag is modified to enforce correct no-slip conditions for obstacles moving with the obstacle velocity $\boldsymbol{u}_s(\boldsymbol{x},t)$. The fluid stress tensor $\boldsymbol{\sigma}(\boldsymbol{u},p)$ and strain rate tensor $\boldsymbol{\epsilon}(\boldsymbol{u},p)$ are defined as follows:

$$\boldsymbol{\sigma}(\boldsymbol{u},p) = -p\boldsymbol{I} + 2\mu\,\boldsymbol{\epsilon}(\boldsymbol{u},p), \tag{7}$$

$$\boldsymbol{\epsilon}(\boldsymbol{u},p) = \frac{1}{2}\left(\nabla\boldsymbol{u} + (\nabla\boldsymbol{u})^\top\right). \tag{8}$$

For $\boldsymbol{h} = \mathbf{0}$, (4) is known as a directional do-nothing boundary condition [*S9*, *S34*], where $\boldsymbol{n}$ is the outward normal of the fluid domain, $\beta \le \frac{1}{2}$ is a positive constant and (9) is added for backflow stabilization with

$$((\boldsymbol{u}-\boldsymbol{w})\cdot\boldsymbol{n})_- := \frac{1}{2}((\boldsymbol{u}-\boldsymbol{w})\cdot\boldsymbol{n} - |(\boldsymbol{u}-\boldsymbol{w})\cdot\boldsymbol{n}|). \tag{9}$$

The ALE domain $\Omega(t)$ is split up into three time dependent sub-domains by means of the permeability $K(t,\boldsymbol{x})$, namely the fluid sub-domain $\Omega_f(t)$, the porous sub-domain $\Omega_p(t)$ and the solid sub-domain $\Omega_s(t)$.

$$K(t,\boldsymbol{x}) = \begin{cases} K_f \longrightarrow +\infty & \text{if } \boldsymbol{x} \in \Omega_f(t) \\ K_p & \text{if } \boldsymbol{x} \in \Omega_p(t) \\ K_s \longrightarrow 0^+ & \text{if } \boldsymbol{x} \in \Omega_s(t) \end{cases} \tag{10}$$

In $\Omega_f(t)$ the classical ALE-Navier–Stokes equations are recovered, while in Ω_p the full ALE-NSB equations describe fluid flowing trough a moving porous medium, $\boldsymbol{u}$ and p are understood in an averaged sense in this context. In $\Omega_s(t)$ the velocity $\boldsymbol{u}$ is approaching $\boldsymbol{u}_s$ and thus asymptotically satisfying the no-slip condition on the $\Omega_f(t)/\Omega_s(t)$ interface. Note that even in the case where $K \longrightarrow 0^+$ the penalization term has a well defined limit, see [*S1*].

S.I.A. Hemodynamic Afterload Models

Modeling of afterload for hemodynamics is modeled by using a 0D Windkessel model. This means we define $\boldsymbol{h}$ in (4) as

$$\boldsymbol{h} := -p_{\mathrm{WK}}(t)\boldsymbol{n}$$

with the *Windkessel pressure* p_{WK} is governed by the differential algebraic system [*S16*]

$$C_{\mathrm{WK}}\frac{\mathrm{d}}{\mathrm{d}t}p_{\mathrm{d}}(t) + \frac{p_{\mathrm{d}}(t)}{R_{\mathrm{WK}}} = Q(t), \tag{11}$$

$$p_{\mathrm{WK}}(t) = Z_{\mathrm{WK}}Q(t) + p_{\mathrm{d}}(t), \tag{12}$$

$$Q(t) := \int_{\Gamma_{\mathrm{outflow}}} \boldsymbol{u}\cdot\boldsymbol{n}\,\mathrm{d}s_{\boldsymbol{x}}. \tag{13}$$

In the case of multiple Windkessel outlets we will use the same notation for variables with an added i subscript indicating multiple outlets. Tools for personalization of the individual Windkessel parameters can be found in [*S33*].

S.I.B. Variational Formulation and Numerical Stabilization

Following [*S7*, *S8*] the discrete variational formulation of (1) including the boundary conditions (4), (5) and (3) can be stated in the following abstract form: Find $\boldsymbol{u}^h \in [\mathcal{S}^1_{h,\boldsymbol{g},\Gamma_D}(\mathcal{T}_{\mathrm{N}})]^3$, $p^h \in \mathcal{S}^1_h(\mathcal{T}_{\mathrm{N}})$ such that, for all $\boldsymbol{v}^h \in [\mathcal{S}^1_{h,\mathbf{0},\Gamma_D}(\mathcal{T}_{\mathrm{N}})]^3$ and for all $q^h \in \mathcal{S}^1_h(\mathcal{T}_{\mathrm{N}})$

$$A_{\mathrm{NS}}(\boldsymbol{v}^h, q^h; \boldsymbol{u}^h, p^h) + S_{\mathrm{RBVMS}}(\boldsymbol{v}^h, q^h; \boldsymbol{u}^h, p^h) = F_{\mathrm{NS}}(\boldsymbol{v}_h) \tag{14}$$

with the bilinear form of the NSB equations

$$\begin{aligned} &A_{\mathrm{NS}}(\boldsymbol{v}^h, q^h; \boldsymbol{u}^h, p^h) = \\ &\int_{\Omega} \rho\,\boldsymbol{v}^h \cdot \left[\left(\frac{\partial \boldsymbol{u}^h}{\partial t} + (\boldsymbol{u}^h - \boldsymbol{w})\cdot\nabla\boldsymbol{u}^h + \frac{\nu}{K}(\boldsymbol{u}^h - \boldsymbol{u}^h_s)\right) + \varepsilon(\boldsymbol{v}^h) : \boldsymbol{\sigma}(\boldsymbol{u}^h, p^h)\right]\,\mathrm{d}\boldsymbol{x} \\ &- \int_{\Gamma_{\mathrm{outflow}}} \rho\beta((\boldsymbol{u}^h - \boldsymbol{w})\cdot\boldsymbol{n})_- \boldsymbol{v}^h\cdot\boldsymbol{u}^h\,\mathrm{d}s_{\boldsymbol{x}} + \int_{\Omega} q^h\nabla\cdot\boldsymbol{u}^h\,\mathrm{d}\boldsymbol{x}, \end{aligned} \tag{15}$$

the bilinear form S_{RBVMS}, which will be explained later in Equation (21), and the right hand side contribution

$$F_{\text{NS}} = -p_{\text{WK}} \int_{\Gamma_{\text{outflow}}} \boldsymbol{n} \cdot \boldsymbol{v}_h \, \mathrm{d}s_{\boldsymbol{x}}. \tag{16}$$

We use standard notation to describe the finite element function space $[\mathcal{S}^1_{h,\boldsymbol{g},\Gamma_D}(\mathcal{T}_N)]^3$ as a conformal vector-valued trial space of piece-wise linear, globally continuous basis functions $\boldsymbol{v}_h$ over a tessellation $\mathcal{T}_N$ of Ω into N finite elements constrained by $\boldsymbol{v}_h = \boldsymbol{g}$ on Dirichlet boundaries, more precisely

$$[\mathcal{S}^1_{h,\boldsymbol{g},\Gamma_D}(\mathcal{T}_N)]^3 := \left\{\boldsymbol{v}_h \in [C(\mathcal{T}_N)]^3 : \boldsymbol{v}_h|_{\tau_l} \in [\mathbb{P}^1(\tau_l)]^3 \, \forall \tau_l \in \mathcal{T}_N \, \wedge \, \boldsymbol{v}_h|_{\Gamma_D} = \boldsymbol{g}\right\},$$
$$\mathcal{S}^1_h(\mathcal{T}_N) := \left\{q_h \in C(\mathcal{T}_N) : q_h|_{\tau_l} \in \mathbb{P}^1(\tau_l) \, \forall \tau_l \in \mathcal{T}_N\right\},$$

where for this work $\Gamma_D := \Gamma_{\text{noslip}} \cup \Gamma_{\text{inflow}}$. For further details we refer to [*S10*, *S40*]. As previously described in [*S26*] we utilize the residual based variational multiscale (RBVMS) formulation as proposed in [*S7*, *S8*], providing turbulence modeling in addition to numerical stabilization. In the following we give a short summary of the changes necessary to use RBVMS methods for the ALE-NSB equations. Briefly, the RBVMS formulation is based on a decomposition of the solution and weighting function spaces into coarse and fine scale subspaces and the corresponding decomposition of the velocity and the pressure and their respective test functions. Henceforth the fine scale quantities and their respective test functions shall be denoted with the superscript $'$. We assume $\boldsymbol{u}_s = \boldsymbol{u}_s^h$, quasi-static fine scales ($\frac{\partial \boldsymbol{u}'}{\partial t} = 0$), as well as $\frac{\partial \boldsymbol{v}^h}{\partial t} = 0$, $\boldsymbol{u}' = \boldsymbol{0}$ on $\partial\Omega(t)$ and incompressibility conditions for $\boldsymbol{u}^h$ and $\boldsymbol{u}'$. The fine scale pressure and velocity are approximated in an element-wise manner by means of the residuals $\boldsymbol{r}_M$ and r_C.

$$\boldsymbol{u}' = -\frac{\tau_{\text{SUPS}}}{\rho} \, \boldsymbol{r}_M(\boldsymbol{u}^h, p^h) \tag{17}$$
$$p' = -\rho \, \nu_{\text{LSIC}} \, r_C(\boldsymbol{u}^h) \tag{18}$$

The residuals of the NSB equations and the incompressibility constraint are:

$$\boldsymbol{r}_M(\boldsymbol{u}^h, p^h) = \rho \frac{\partial}{\partial t}\boldsymbol{u}^h + \rho(\boldsymbol{u}^h - \boldsymbol{w}) \cdot \nabla \boldsymbol{u}^h - \nabla \cdot \boldsymbol{\sigma}(\boldsymbol{u}^h, p^h) + \frac{\mu}{K}(\boldsymbol{u}^h - \boldsymbol{u}_s^h) \tag{19}$$
$$r_C(\boldsymbol{u}^h) = \nabla \cdot \boldsymbol{u}^h \tag{20}$$

Taking all assumptions into consideration and employing the scale decomposition followed by partial integration yields the bilinear form of the RBVMS formulation $S_{\text{RBVMS}}(\boldsymbol{v}^h, q^h; \boldsymbol{u}^h, p^h)$,

$$\begin{aligned} &S_{\text{RBVMS}}(\boldsymbol{v}^h, q^h; \boldsymbol{u}^h, p^h) = \\ &+ \sum_{\Omega_e \in \mathcal{T}_N} \int_{\Omega_e} \tau_{\text{SUPS}} \left((\boldsymbol{u}^h - \boldsymbol{w}) \cdot \nabla \boldsymbol{v}^h + \frac{1}{\rho} \nabla q^h - \frac{\nu}{K} \boldsymbol{v}^h \right) \boldsymbol{r}_M(\boldsymbol{u}^h, p^h) \, \mathrm{d}\boldsymbol{x} \\ &+ \sum_{\Omega_e \in \mathcal{T}_N} \int_{\Omega_e} \rho \, \nu_{\text{LSIC}} \, \nabla \cdot \boldsymbol{v}^h \, r_C(\boldsymbol{u}^h) \, \mathrm{d}\boldsymbol{x} \\ &- \sum_{\Omega_e \in \mathcal{T}_N} \int_{\Omega_e} \tau_{\text{SUPS}} \, \boldsymbol{v}^h \cdot \left(\boldsymbol{r}_M(\boldsymbol{u}^h, p^h) \cdot \nabla \boldsymbol{u}^h \right) \mathrm{d}\boldsymbol{x} \\ &- \sum_{\Omega_e \in \mathcal{T}_N} \int_{\Omega_e} \frac{\tau_{\text{SUPS}}^2}{\rho} \nabla \boldsymbol{v}^h : (\boldsymbol{r}_M(\boldsymbol{u}^h, p^h) \otimes \boldsymbol{r}_M(\boldsymbol{u}^h, p^h)) \, \mathrm{d}\boldsymbol{x}. \end{aligned} \tag{21}$$

The residuals (19) and (20) are evaluated for every element $\Omega_e \in \mathcal{T}_N$. Following [*S37*] the stabilization parameters τ_{SUPS} and ν_{LSIC} are defined as:

$$\tau_{\mathrm{SUPS}} := \left(\frac{4}{\Delta t^2} + (\boldsymbol{u}^h - \boldsymbol{w}) \cdot \boldsymbol{G}(\boldsymbol{u}^h - \boldsymbol{w}) + \left(\frac{\nu}{K}\right)^2 + C_I \nu^2 \boldsymbol{G} : \boldsymbol{G} \right)^{-\frac{1}{2}} \tag{22}$$

$$\nu_{\mathrm{LSIC}} := \frac{1}{\mathrm{tr}(\boldsymbol{G})\, \tau_{\mathrm{SUPS}}} \tag{23}$$

Here $\boldsymbol{G}$ is the three dimensional element metric tensor defined per finite element as

$$\boldsymbol{G}|_{\tau_l} := \boldsymbol{J}_l^{-1} \boldsymbol{J}_l^{-\top},$$

with $\boldsymbol{J}_l$ being the Jacobian of the transformation of the reference element to the physical finite element $\tau_l \in \mathcal{T}_N$, Δt denotes time step size and C_I is a positive constant, taken as 30, derived from an element-wise inverse estimate. For further details see [*S7*, *S8*].

S.I.C. Numerical Solution Strategy

Spatio-temporal discretization of all PDEs and the solution of the arising systems of equations relied upon the Cardiac Arrhythmia Research Package (CARPentry), see [*S43*]. For temporal discretization of the ALE-NSB equations we used the generalized-α method, see [*S23*] with a spectral radius $\rho_\infty = 0.2$. For updating the Windkessel pressures p_{WK} we discretized (11) with an implicit Euler method. For ease of coupling with our CFD solver the calculation of $Q(t)$ in (13) is lagged by one Newton iteration. After discretization in space as described in Section S.I.B and temporal discretization using the generalized-α integrator we obtain a nonlinear algebraic system to solve for advancing time from timestep t_n to t_{n+1}. A quasi inexact Newton-Raphson method is used to solve this system with linearization approach similar to [*S8*] adapted to the NSB equations. At each iteration a block system of the form

$$\begin{bmatrix} \boldsymbol{K}_h & \boldsymbol{B}_h \\ \boldsymbol{C}_h & \boldsymbol{D}_h \end{bmatrix} \begin{bmatrix} \Delta \boldsymbol{u} \\ \Delta \boldsymbol{p} \end{bmatrix} = - \begin{bmatrix} -\boldsymbol{R}_{\mathrm{upper}} \\ -\boldsymbol{R}_{\mathrm{lower}} \end{bmatrix},$$

is solved with $\boldsymbol{K}_h$, $\boldsymbol{B}_h$, $\boldsymbol{C}_h$, and $\boldsymbol{D}_h$ denoting the Jacobian matrices, $\Delta\boldsymbol{u}$, $\Delta\boldsymbol{p}$ representing the velocity and pressure updates and $\boldsymbol{R}_{\mathrm{upper}}$, $\boldsymbol{R}_{\mathrm{lower}}$ indicating the residual contributions. In this regard we use the flexible generalized minimal residual method (fGMRES) and efficient preconditioning based on the `PCFIELDSPLIT`[1] package from the library *PETSc* [*S4–S6*] and the incorporated suite *HYPRE BoomerAMG* [*S19*]. By extending our previous work [*S3*, *S26*, *S27*] we implemented the methods in the finite element code *Cardiac Arrhythmia Research Package* (CARPentry) [*S42*, *S43*].

S.II. Obstacle Representation

Here we want to give a brief description of how we represent moving obstacles for usage in the ALE-NSB equations. This task is solved by representing obstacles using triangular surface meshes followed by element-wise calculation of the partial volume covered by the obstacle. In the first step, all nodes within the obstacle are identified using the ray casting algorithm [*S18*, *S35*]. Subsequently, all elements are split into three categories and receive a corresponding volume fraction value v_f, describing the partial volume covered by the obstacle:

- Elements fully covered by the obstacle lie in Ω_s, consequently $v_f = 1$.
- Elements outside the obstacle lie in Ω_f and obtain $v_f = 0$.
- Elements that are split by the element surface correspond to elements in Ω_p, hence

$$v_f = \frac{V_{in}}{V_{tot}} \tag{24}$$

[1] `https://www.mcs.anl.gov/petsc/petsc-current/docs/manualpages/PC/PCFIELDSPLIT.html`

where V_{in} denotes the element volume covered by the obstacle and V_{tot} is the total element volume.

This procedure is carried out for every time step and yields a time-dependent, element-based volume fraction distribution $v_f(t, \boldsymbol{x})$, that serves as a basis to provide a suitable permeability distribution, see Figure S1. In this work we define $\frac{1}{K(t,\boldsymbol{x})} := \frac{v_f(t,\boldsymbol{x})}{\kappa}$ with κ being a fixed penalization factor, e.g. $\kappa = 10^{-8}$. All permeability distributions in this work have been generated using the open-source software *Meshtool*[2], see [*S36*] and [*S17*] for algorithmic details. In the case of obstacles that change from open to closed state over time we use a simple scaling function. For example, assume an obstacle representing a heart valve region will be open at a time instance t_{op} and it takes $\mathrm{dur}_{\mathrm{V}}$ time to switch from open to closed we define

$$\chi(t) := \begin{cases} \frac{t_{\mathrm{op}}-t}{\mathrm{dur}_{\mathrm{V}}} & t \in [t_{\mathrm{op}} - \mathrm{dur}_{\mathrm{V}}, t_{\mathrm{op}}] \\ 0 & t \in [t_{\mathrm{op}}, t_{\mathrm{cl}} - \mathrm{dur}_{\mathrm{V}}] \\ \frac{t-t_{\mathrm{cl}}+\mathrm{dur}_{\mathrm{V}}}{\mathrm{dur}_{\mathrm{V}}} & t \in [t_{\mathrm{cl}} - \mathrm{dur}_{\mathrm{V}}, t_{\mathrm{cl}}] \\ 1 & \text{else} \end{cases}, \tag{25}$$

and modify $\frac{1}{K(t,\boldsymbol{x})}$ to $\frac{\chi}{K(t,\boldsymbol{x})}$.

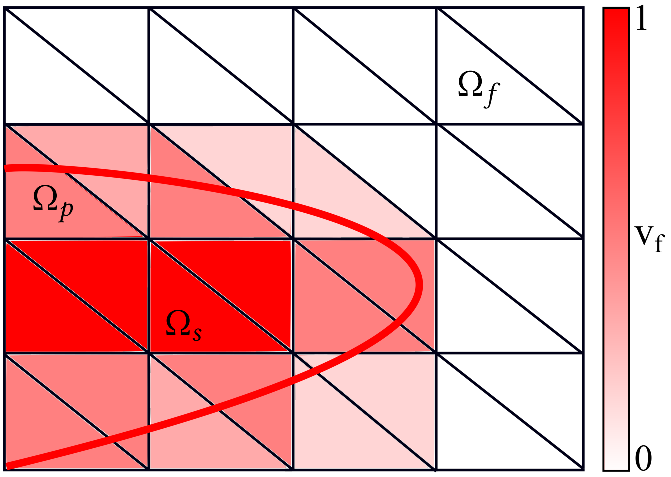

Figure S1: Schematic representation of the v_f distribution associated to an obstacle,which is represented by the red line, at a fixed point time t.

S.III. Computation of Residence Times on Moving Domains

Here we will give a brief outline of the methods and algorithms used to compute residence time distributions. The starting point is the following PDE describing the time evolution of a residence time distribution field originating from [*S14*, *S31*]. Given a moving fluid domain $\Omega(t) \subset \mathbb{R}^3$ [*s*] and a region of interest $V(t) \subset \Omega(t)$ the evolution of the time $\tau(t, \boldsymbol{x})$ spent in V by an arbitrarily small fluid particle

[2] `https://bitbucket.org/aneic/meshtool/src/master/`

caught at point $\boldsymbol{x} \in \Omega(t)$ at time t can be described as

$$\begin{aligned}
\frac{\partial}{\partial t}\tau + (\boldsymbol{u} - \boldsymbol{w}) \cdot \nabla\tau - \varepsilon\Delta\tau &= H(t, \boldsymbol{x}) && \text{in } \Omega(t), \\
\frac{\partial}{\partial \boldsymbol{n}}\tau &= 0 && \text{on } \Gamma_{\mathrm{N}}(t), \\
\tau &= g && \text{on } \Gamma_{\mathrm{D}}(t), \\
H(t, \boldsymbol{x}) &:= \begin{cases} 1 & \text{if } (t, \boldsymbol{x}) \in V(t) \\ 0 & \text{else} \end{cases},
\end{aligned}$$

with the fluid velocity $\boldsymbol{u}$, the ALE mesh velocity $\boldsymbol{w}$ and a small artificial diffusion parameter ε $[\mathrm{m}^2\mathrm{s}^{-1}]$ added for numerical stability of the method. For this work we have used $\varepsilon = 1 \times 10^{-12}$. The fluid velocity $\boldsymbol{u}$ as well as the ALE mesh velocity $\boldsymbol{w}$ are assumed to be given functions, e.g. coming from a pre-computed CFD simulation. In our applications we set $\Gamma_D(t) = \emptyset$ and $\Gamma_N(t) = \partial\Omega(t)$. Furthermore, the region of interest $V(t)$ is assumed as a time-evolving tag region assigned to a particular anatomic region, e.g.: ventricular blood pools, and left atrial appendage respectively. After discretization we have

$$\boldsymbol{M}_h(t)\dot{\boldsymbol{\tau}} + \boldsymbol{K}_h(t)\boldsymbol{\tau}(t) = \boldsymbol{F}_h(t) \tag{26}$$

with

$$\begin{aligned}
\boldsymbol{M}_{h,ij}(t) &= \int\limits_{\Omega(t)} \phi_i(t)\phi_j(t)\,\mathrm{d}x, \\
\boldsymbol{K}_{h,ij}(t) &= \varepsilon \int\limits_{\Omega(t)} \nabla\phi_i(t) \cdot \nabla\phi_j(t)\,\mathrm{d}x \\
&\quad + \int\limits_{\Omega(t)} (\boldsymbol{u}(t) - \boldsymbol{w}(t)) \cdot \nabla\phi_i(t)\phi_j(t)\,\mathrm{d}x, \\
\boldsymbol{F}_{h,j}(t) &= \int\limits_{V(t)} \phi_j(t)\,\mathrm{d}x,
\end{aligned}$$

with $\{\phi_i(t)\}_{i=0}^n$ being the time-dependent test and trials functions in the ALE domain. For regular domain movement it is safe to assume that $\boldsymbol{M}_h(t)$ is invertible for all t and we can rewrite (26) as

$$\dot{\boldsymbol{\tau}} + \boldsymbol{M}_h^{-1}(t)\boldsymbol{K}_h(t)\boldsymbol{\tau}(t) = \boldsymbol{M}_h^{-1}(t)\boldsymbol{F}_h(t). \tag{27}$$

Next, we apply the modified Crank-Nicholson scheme in time as proposed in [*S15*] giving

$$\left(\boldsymbol{M}_C + \frac{\Delta t}{2}\boldsymbol{K}\right)\boldsymbol{\tau}^{n+1} = \left(\boldsymbol{M}_C - \frac{\Delta t}{2}\boldsymbol{K}\right)\boldsymbol{\tau}^n + \frac{\Delta t}{2}\boldsymbol{F}, \tag{28}$$

where we used the following shorthand notation

$$\begin{aligned}
\boldsymbol{M}_{\mathrm{C}} &:= \boldsymbol{M}_h(t^{n+\frac{1}{2}}), \\
\boldsymbol{K} &:= \boldsymbol{K}_h(t^{n+\frac{1}{2}}), \\
\boldsymbol{F} &:= \boldsymbol{F}_h(t^{n+\frac{1}{2}}).
\end{aligned}$$

Equation (28) is our starting point for applying the FCT scheme similar to [*S25*]. Following the ideas of FEM-FCT methods we define the matrices

$$\begin{aligned}
\boldsymbol{L} &:= \boldsymbol{K} + \boldsymbol{D}, \\
\boldsymbol{D}_{ij} &:= \begin{cases} -\max\left\{0, \boldsymbol{K}_{ij}, \boldsymbol{K}_{ji}\right\} & \text{if } i \neq j, \\ -\sum_{j=1, j\neq i}^{N} \boldsymbol{D}_{ij} & \text{else} \end{cases}, \\
\boldsymbol{M}_{\mathrm{L}} &:= \operatorname{diag}(\boldsymbol{m}), \\
\boldsymbol{m}_i &:= \sum_{j=1}^{N} \boldsymbol{M}_{\mathrm{C},ij}.
\end{aligned}$$

The construction of $\boldsymbol{L}$ ensures zero row and column sums. Instead of (28) we consider

$$\left(\boldsymbol{M}_L + \frac{\Delta t}{2}\boldsymbol{L}\right)\boldsymbol{\tau}^{n+1} = \left(\boldsymbol{M}_L - \frac{\Delta t}{2}\boldsymbol{L}\right)\boldsymbol{\tau}^{n} + \frac{\Delta t}{2}\boldsymbol{F}, \tag{29}$$

which represents a stable low-order scheme whose solution doesn't possess any over or undershoots but suffers from to smeared layers. To correct this behavior and artificial flux correction vector $\boldsymbol{f}^*(\tau^{n+1}, \tau^n)$ is added to the right hand side of (29). The definition of $\boldsymbol{f}^*$ follows from an ad-hoc ansatz

$$\boldsymbol{f}_i^*(\tau^{n+1}, \tau^n) = \sum_{j=1}^{n} \alpha_{ij} r_{ij},$$

with the *fluxes* r_{ij} defined as

$$\begin{aligned}
r_{ij} := {} & \boldsymbol{M}_{C,ij}(\boldsymbol{\tau}_i^{n+1} - \boldsymbol{\tau}_j^{n+1}) - \boldsymbol{M}_{C,ij}(\boldsymbol{\tau}_i^{n} - \boldsymbol{\tau}_j^{n}) \\
& - \frac{\Delta t}{2}\boldsymbol{D}_{ij}(\boldsymbol{\tau}_i^{n+1} - \boldsymbol{\tau}_j^{n+1}) - \frac{\Delta t}{2}\boldsymbol{D}_{ij}(\boldsymbol{\tau}_i^{n} - \boldsymbol{\tau}_j^{n}).
\end{aligned} \tag{30}$$

and weights $\alpha_{ij} \in [0, 1]$. The representation for r_{ij} follows from first subtracting (28) from (29) and applying the properties of the matrices $\boldsymbol{M}_C$ and $\boldsymbol{D}$. This formulation represents a nonlinear system. In [*S25*] a linear variant has been proposed which we adapted to the moving-domain case. For this we use the explicit solution $\tilde{\boldsymbol{\tau}}$ to (29), by means of an explicit Euler scheme approximating the solution $\boldsymbol{\tau}^{n+\frac{1}{2}}$ at time step $t_n + \frac{\Delta t}{2}$, reading

$$\tilde{\boldsymbol{\tau}} := \boldsymbol{\tau}^n - \frac{\Delta t}{2}\boldsymbol{M}_L^{-1}\left(\boldsymbol{L}\boldsymbol{\tau}^n - \boldsymbol{F}\right).$$

Inserting $\tilde{\boldsymbol{\tau}}$ into (30) and rearranging terms yields

$$r_{ij} = \Delta t\left(\boldsymbol{M}_{C,ij}(\boldsymbol{\eta}_i^{n+\frac{1}{2}} - \boldsymbol{\eta}_j^{n+\frac{1}{2}}) - \boldsymbol{D}_{ij}(\tilde{\boldsymbol{\tau}}_i - \tilde{\boldsymbol{\tau}}_j)\right),$$

where $\boldsymbol{\eta}_i^{n+\frac{1}{2}} := (\boldsymbol{M}_L^{-1}(\boldsymbol{F} - \boldsymbol{L}\boldsymbol{\tau}^n))_i$. Additionally, as suggested in [*S29*], we employ prelimiting in the form

$$r_{ij} = 0 \quad \text{if } r_{ij}(\tilde{\boldsymbol{\tau}}_i - \tilde{\boldsymbol{\tau}}_j) < 0.$$

The computation of the weights α_{ij} follows the same procedure as in [*S25*] using Zalesak's algorithm [*S44*]. We also refer to [*S29*, *S30*] for a more detailed overview of the presented method. Computation of the residence time distribution fields have been included as addon in *CARPentry*. After computation of the residence time distribution τ we can calculate the residence time RT spend in $V(t)$ over a time period (t_0, t_1) [*S31*] as

$$\begin{aligned}
\mathrm{RT} &:= \frac{1}{(t_1 - t_0)|V|}\int_{t_0}^{t_1}\int_{\Omega(t)} \tau(t, \boldsymbol{x})H(t, \boldsymbol{x})\,\mathrm{d}\boldsymbol{x}\mathrm{d}t, \\
|V| &:= \frac{1}{t_1 - t_0}\int_{t_0}^{t_1}\int_{\Omega(t)} H(t, \boldsymbol{x})\,\mathrm{d}\boldsymbol{x}\mathrm{d}t.
\end{aligned}$$

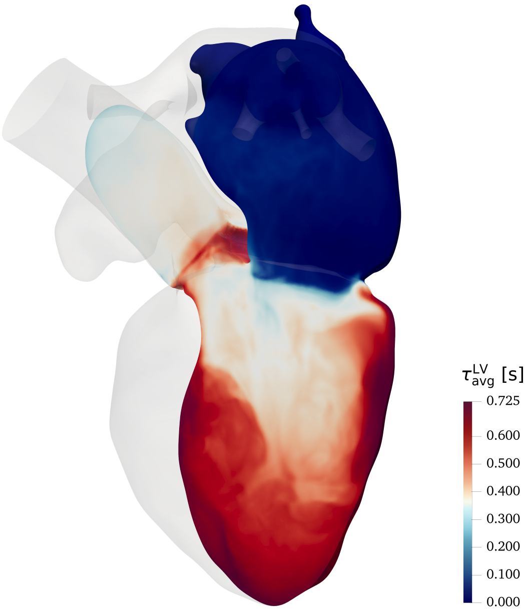

(a) $V(t)$ defined as tag region of left ventricular bloodpool.

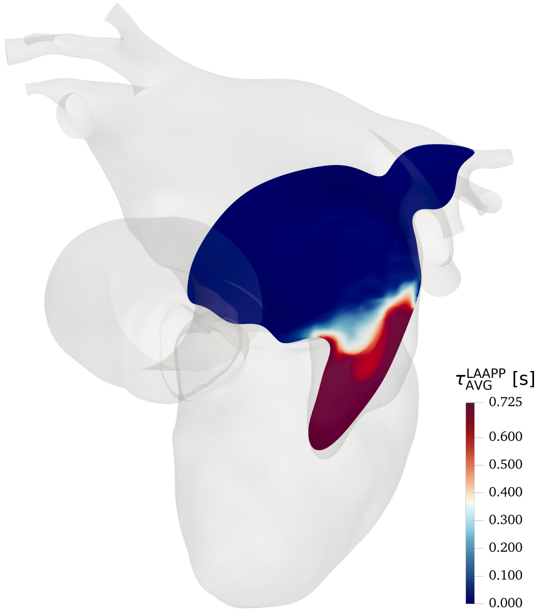

(b) $V(t)$ defined as tag region of left atrial appendage.

Figure S2: Time-averaged residence time distributions τ_{AVG} with $V(t)$ defined through different labels in the computational mesh. Time average taken over the final two heartbeats with beatlength equal to 0.725 s.

Figure S2 and Figure S3 show illustrations of time averaged residence time distributions that were generated for this work as part of the sensitivity analysis.

S.IV. Pope's Criterion of Turbulence Resolution

In LES type formulations the resolved velocity field is fundamentally linked to the numerical method used, hence there is no notion of convergence to the solution of a partial differential equation [*S38*]. This leads to the problem that mesh convergence often cannot be established by the classical methods [*S12*, *S21*, *S24*, *S32*, *S39*]. To remedy this problem [*S38*] proposes the use of a measure of turbulence resolution M, see (32), utilizing the fraction of turbulent kinetic energy resolved by the grid in question. In order to obtain a point-wise measure, rather than the kinetic energy itself, the kinetic energy density $K(\boldsymbol{x}, t)$ is considered:

$$K(\boldsymbol{x}, t) = \frac{\rho}{2}\boldsymbol{u}(\boldsymbol{x}, t)^2. \tag{31}$$

The resulting point-wise measure of turbulence resolution M reads:

$$M(\boldsymbol{x}, t) = \frac{K'(\boldsymbol{x}, t)}{K_{tot}(\boldsymbol{x}, t)}. \tag{32}$$

Here K' is the turbulent kinetic energy of the residual motions, hence of the motions not resolved by the grid, and K_{tot} stands for the total kinetic energy. K_{tot} may be written as the sum of the resolved turbulent kinetic energy K^h and the not resolved turbulent kinetic energy K':

$$K_{tot}(\boldsymbol{x}, t) = K^h(\boldsymbol{x}, t) + K'(\boldsymbol{x}, t) \tag{33}$$

The resolved turbulent kinetic energy K^h is calculated from (31) using the fluctuating part of the resolved fluid velocity $\boldsymbol{u}_f$, which is given by:

$$\boldsymbol{u}_f(\boldsymbol{x}, t) = \overline{\boldsymbol{u}^h}(\boldsymbol{x}, t) - \boldsymbol{u}^h(\boldsymbol{x}, t), \tag{34}$$

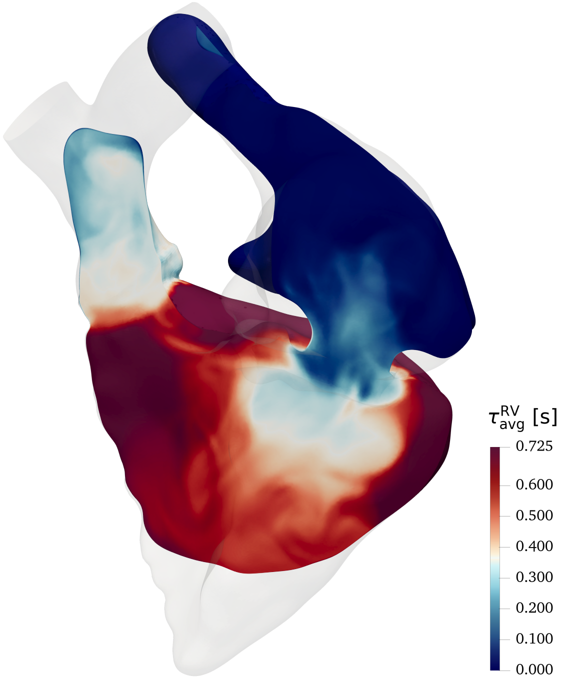

Figure S3: Time-averaged residence time distributions $\tau^{\mathrm{RV}}_{\mathrm{AVG}}$ with $V(t)$ defined as right ventricular blood pool in the computational mesh. Time average taken over the final two heartbeats with beatlength equal to 0.725 s.

where $\overline{\boldsymbol{u}^h}$ is an average with respect to time. When considering a constant inflow $\overline{\boldsymbol{u}^h}$ is given by the standard mean over all time steps ($t = 1 \dots T$, hence $\overline{\boldsymbol{u}^h}$ is not time-dependent) :

$$\overline{\boldsymbol{u}^h}(\boldsymbol{x}) = \frac{1}{T}\sum_{t=1}^{T}\boldsymbol{u}^h(\boldsymbol{x},t) \tag{35}$$

In the case of a pulsatile behavior however a phase average is considered:

$$\overline{\boldsymbol{u}^h}(\boldsymbol{x},t) = \frac{1}{n}\sum_{k=0}^{n-1}\boldsymbol{u}^h(\boldsymbol{x},t+k\tau), \tag{36}$$

where n is the number of cycles or beats and τ is the period or beat length. By the use of (32) a criterion for sufficient mesh resolution is given:

$$M(\boldsymbol{x},t) \le \epsilon_M \tag{37}$$

In [*S38*] a value of $\epsilon_M = 0.2$ is proposed, which corresponds to requiring a minimum of 80% of the total turbulence energy to be resolved.

We calculated this criterion based on the simulation of four beats for different spatial and temporal resolutions with average edge lengths of $\Delta x = 3000, 1500, 750$ μm and time step sizes of $\Delta t = 1450, 725, 362.5$ ms for the mesh of the left heart. Figure S4 shows a comparison of the average Pope criterion, with average taken over all elements of the mesh. From this we concluded that the mesh with average edge length of $\Delta x = 750$ μm and a time step size $\Delta t = 362.5$ ms was sufficient to resolve the flow dynamics in the left heart. For reasons of practicality we used the same average edge length and time step size for the right side heart.

S.V. Input Parameter Variance Effect on Output Features

This serves as additional interpretation for the results in the main manuscript. Figure S5 shows the extracted temporal signals of all parameter sets for the pressure differences $\Delta p_{\mathrm{MV}1,2,3,4}$ in the LA. While

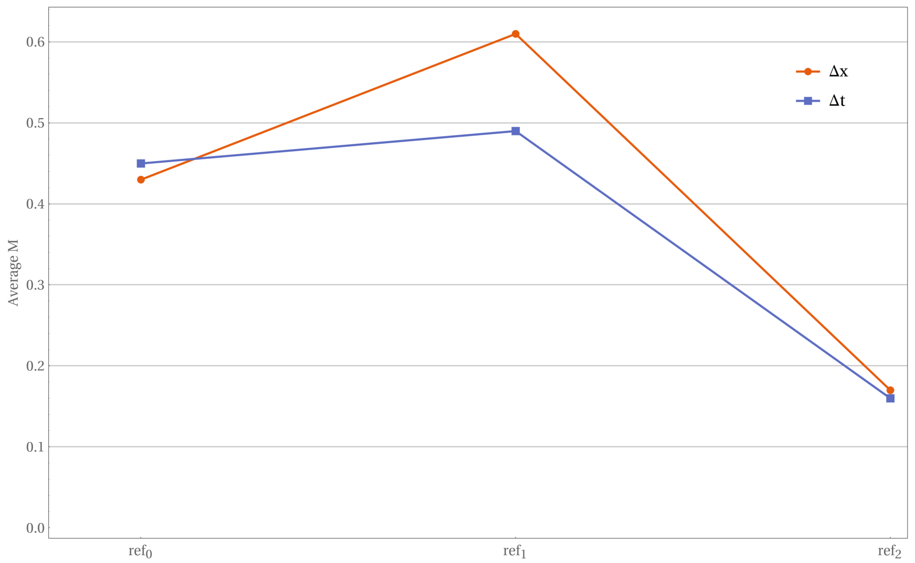

Figure S4: Average Pope criterion for the different spatial and temporal discretization levels. The average is taken over all elements in the mesh.

there is no strong influence on the output in systole, one can see a clear variation in the outputs in diastole.

S.VI. 5-Fold Cross-Validation Results

Table S1 and Table S2 summarize the results of the cross-validation. Features with an average R_2-score of < 0.5 were excluded from global sensitivity analysis in this work. It can be observed that the R_2 scores are on average lower for left heart features. We attribute that to the increased turbulence in left heart blood flow compared to right heart blood flow.

S.VII. Comparing Influence of Time Step Size on Main Vortex Center in the Left Ventricle

To study the influence of time step size on the accuracy of vortex formation we ran a simulation of left heart blood flow with successive refined time step sizes $\Delta t = 362.5,\ 181.25,\ 90.625$ ms. It is known, see [*S28*] and references therein, that vorticity is not a suitable criterion for vortex identification, hence we used used the scaled Q-criterion Q_s defined as

$$
\begin{aligned}
Q_s &:= \frac{1}{2}\left(\frac{\|\Omega\|_\mathrm{F}^2}{\|\boldsymbol{S}\|_\mathrm{F}^2} - 1\right), \\
Q &:= \|\boldsymbol{S}\|_F^2 Q_s, \\
\Omega &:= \frac{1}{2}\left(\nabla \boldsymbol{u} - \nabla \boldsymbol{u}^\top\right), \\
\boldsymbol{S} &:= \frac{1}{2}\left(\nabla \boldsymbol{u} + \nabla \boldsymbol{u}^\top\right), \\
\|\boldsymbol{A}\|_\mathrm{F}^2 &:= \sum_{i,j=1}^{3} A_{ij}^2.
\end{aligned}
\tag{38}
$$

The difference to the standard Q-criterion is the normalization w.r.t. strain, making it easier to find adequate thresholds for visualization. We used the threshold value of 2.5 and calculated the isosurfaces

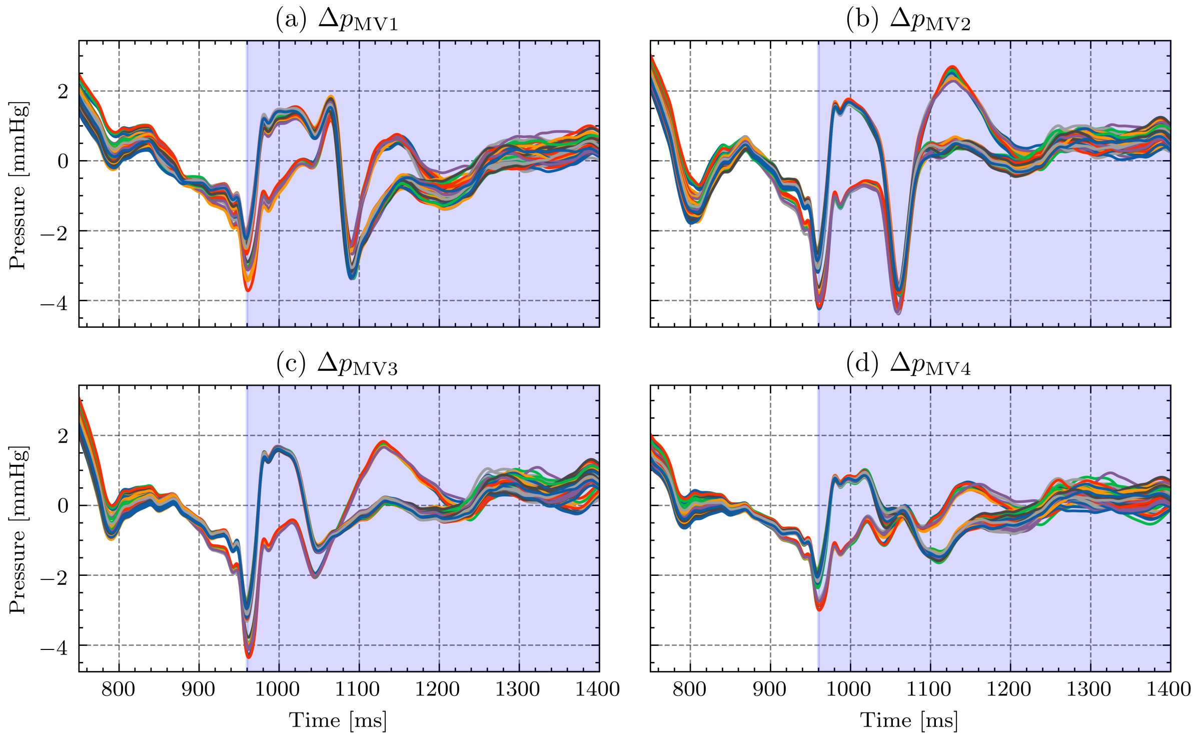

Figure S5: Extracted time signals for pressure differences in the LA for the second heart beat. Diastole is indicated by the shaded blue area in the plots.

Table S1: R_2-Scores for cross validation of LV features.

Feature	R_2-Scores					Average
	Fold 1	Fold 2	Fold 3	Fold 4	Fold 5	
Δp_{AV}	0.96	0.97	0.95	0.96	0.87	0.94
Δp_{MV}	0.89	0.97	0.96	0.96	0.89	0.93
Δp_{MV1}	0.57	0.47	0.61	0.49	0.53	0.53
Δp_{MV2}	0.31	0.41	0.48	0.42	0.49	0.42
Δp_{MV3}	0.34	0.05	0.52	0.30	0.05	0.25
Δp_{MV4}	0.45	0.51	0.48	0.48	0.61	0.51
Δp_{AMV}	0.49	0.66	0.45	0.55	0.56	0.54
Δp_{AAV}	0.19	0.44	0.65	0.91	0.56	0.55
Δp_{AVMV}	0.63	0.72	0.65	0.82	0.63	0.69
$E_{\mathrm{k,LV}}$	0.71	0.72	0.81	0.86	0.90	0.80
$E_{\mathrm{k,AO}}$	0.68	0.68	0.73	0.72	0.67	0.70
$E_{\mathrm{k,LA}}$	0.92	0.90	0.79	0.88	0.89	0.88
$\mathrm{RT}_{\mathrm{LV}}$	0.39	0.73	0.27	0.61	0.53	0.51
$\mathrm{RT}_{\mathrm{LAAPP}}$	0.83	0.89	0.79	0.89	0.87	0.85
$\mathrm{maxv}_{\mathrm{AV}}$	0.85	0.89	0.74	0.80	0.85	0.83
$\mathrm{maxv}_{\mathrm{MV}}$	0.46	0.71	0.06	-0.18	-0.15	0.18

Table S2: R_2-Scores for cross validation of RV features.

Feature	R_2-Scores					Average
	Fold 1	Fold 2	Fold 3	Fold 4	Fold 5	
Δp_{PV}	0.99	0.99	0.99	0.98	0.96	0.98
Δp_{TV}	0.96	0.95	0.91	0.94	0.95	0.94
Δp_{TV1}	0.97	0.97	0.97	0.98	0.98	0.97
Δp_{TV2}	0.98	0.94	0.97	0.99	0.98	0.97
Δp_{TV3}	0.96	0.94	0.95	0.97	0.97	0.96
Δp_{ATV}	0.78	0.90	0.93	0.81	0.96	0.88
Δp_{APA}	0.43	0.50	0.92	0.59	0.65	0.62
Δp_{PATV}	0.99	0.99	0.99	0.99	0.99	0.99
$E_{\text{k,RV}}$	0.96	0.96	0.99	0.99	0.99	0.97
$E_{\text{k,PA}}$	0.98	0.98	0.96	0.98	0.98	0.98
$E_{\text{k,RA}}$	0.92	0.96	0.93	0.98	0.92	0.94
RT_{RV}	0.98	0.99	0.91	0.95	0.94	0.95
maxv_{PV}	0.98	0.89	0.93	0.94	0.87	0.92
maxv_{TV}	0.98	0.92	0.91	0.94	0.79	0.91

for this value. Afterwards, we extracted the major vortex component in the left ventricle at peak diastole and calculated the vortex centers for each time discretization. Table S3 shows a comparison of the relative error of the identified vortex centers and Figure S6 shows a visual comparison of the resulting isosurfaces. From this results we concluded that our simulations accurately resolved the vortices at the chosen time step size of $\Delta t = 362.5$ ms coinciding with the results obtained from using Pope's criterion as described in section S.IV.

Table S3: Results for Vortex Center Identification.

Δt [ms]	Vortex Center			rel. Error [%]
	x [mm]	y [mm]	z [mm]	
362.5	-67.40	191.162	1889.86	0.17
181.25	-67.81	190.369	1889.88	0.15
90.625	-70.39	191.572	1890.95	—

S.VIII. Scatterplot of the Locations of Failing Samples

Here we include a projection of the training data set used for calibrating the left heart GPEs overlayed with the samples that led to failed simulations. From the result in Figure S7 we conclude that the failed samples appear to be random samples.

References

[S1] P. Angot, C.-H. Bruneau, and P. Fabrie. A penalization method to take into account obstacles in incompressible viscous flows. *Numerische Mathematik*, 81(4):497–520, Feb 1999. ISSN 0945-3245. doi: 10.1007/s002110050401.

[S2] E. Arquis and J. Caltagirone. Sur les conditions hydrodynamiques au voisinage d'une interface

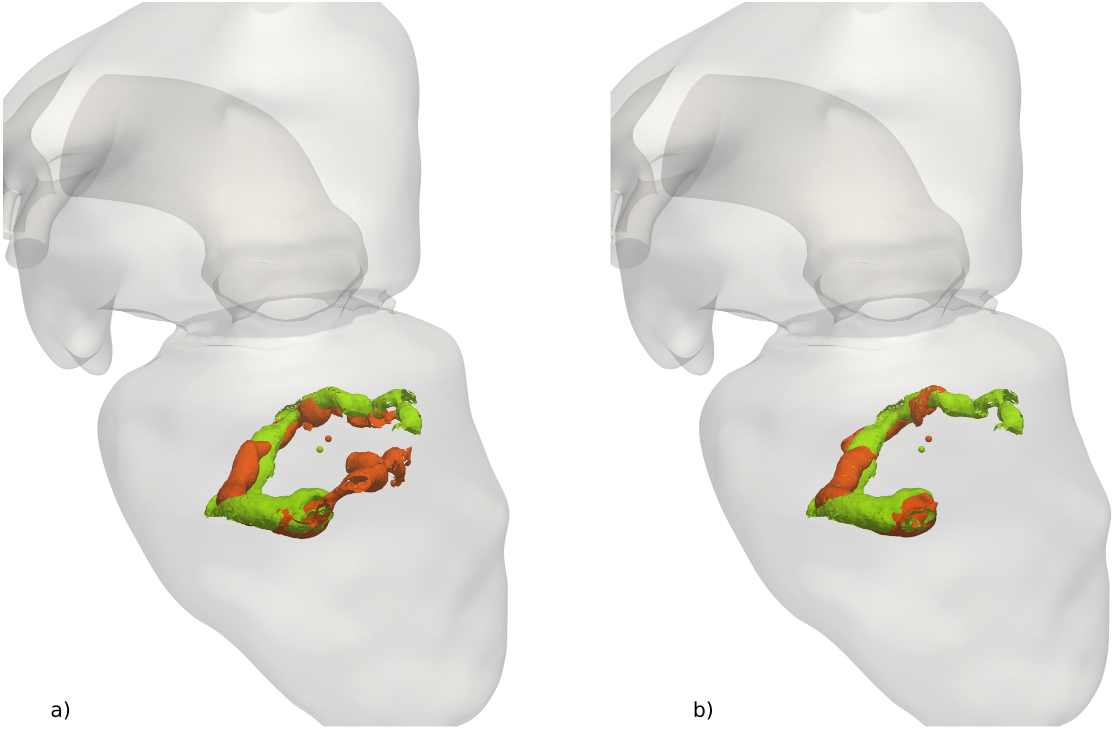

Figure S6: Comparison of the major vortex centers extracted from isosurfaces of the scaled Q-criterion Q_s at value 2.5 at peak diastole for different time discretizations: a) Δt = 362.5ms (orange) and reference time step Δt = 90.625ms (green), and b) Δt = 181.25ms (orange) and reference time step Δt = 90.625ms (green). Additionally the vortex centers are depicted as colored spheres in orange and green.

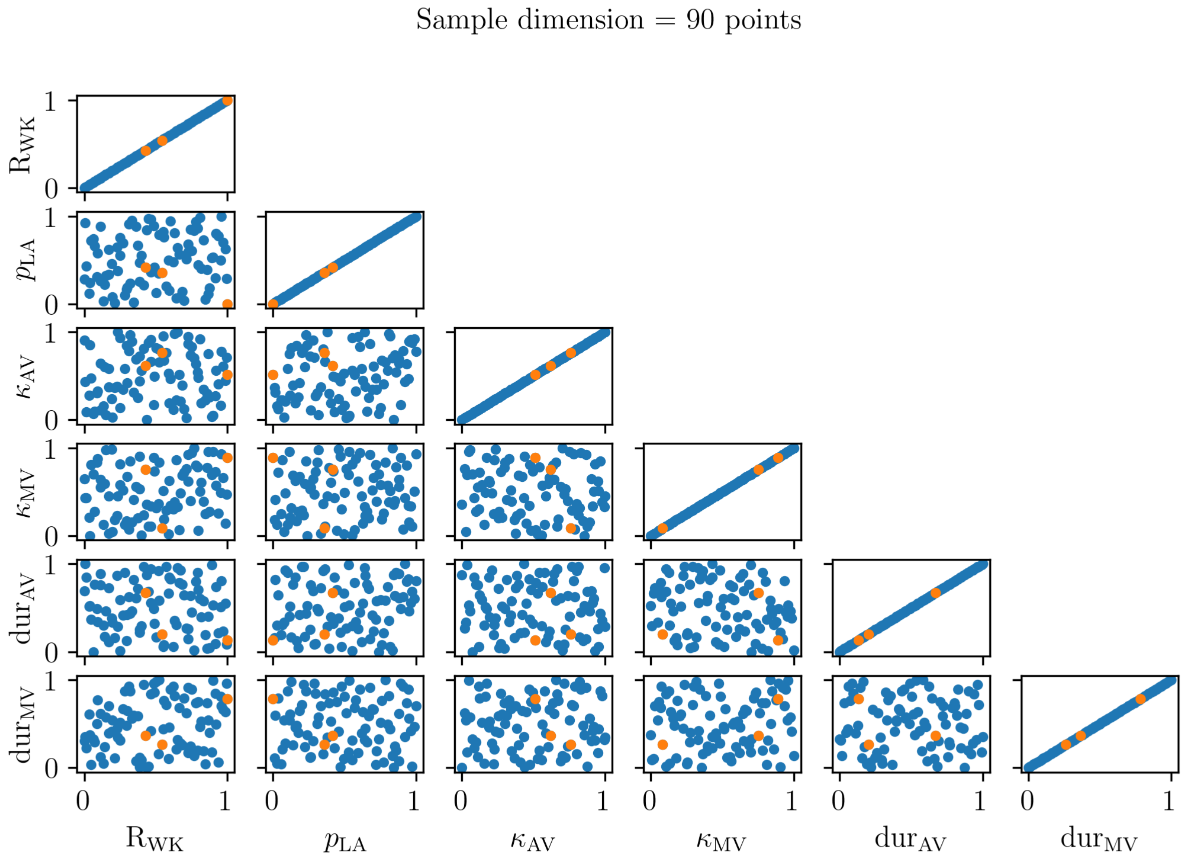

Figure S7: 2D Projection of the Latin Hypercube Sampling used for training the GPEs. Blue markers depict all training points, while orange markers show the samples leading to failed simulations.

milieu fluide-milieu poreux: application a la convection naturelle. *CR Acad. Sci. Paris II*, 299:1–4, 1984.

[S3] C. M. Augustin, A. Neic, M. Liebmann, A. J. Prassl, S. A. Niederer, G. Haase, and G. Plank. Anatomically accurate high resolution modeling of human whole heart electromechanics: A strongly scalable algebraic multigrid solver method for nonlinear deformation. *Journal of Computational Physics*, 305:622–646, Jan. 2016. URL `https://doi.org/10.1016/j.jcp.2015.10.045`.

[S4] S. Balay, W. D. Gropp, L. C. McInnes, and B. F. Smith. Efficient management of parallelism in object oriented numerical software libraries. In E. Arge, A. M. Bruaset, and H. P. Langtangen, editors, *Modern Software Tools in Scientific Computing*, pages 163–202. Birkhäuser Press, 1997.

[S5] S. Balay, S. Abhyankar, M. F. Adams, J. Brown, P. Brune, K. Buschelman, L. Dalcin, A. Dener, V. Eijkhout, W. D. Gropp, D. Karpeyev, D. Kaushik, M. G. Knepley, D. A. May, L. C. McInnes, R. T. Mills, T. Munson, K. Rupp, P. Sanan, B. F. Smith, S. Zampini, H. Zhang, and H. Zhang. PETSc Web page. `https://www.mcs.anl.gov/petsc`, 2019. URL `https://www.mcs.anl.gov/petsc`.

[S6] S. Balay, S. Abhyankar, M. F. Adams, J. Brown, P. Brune, K. Buschelman, L. Dalcin, A. Dener, V. Eijkhout, W. D. Gropp, D. Karpeyev, D. Kaushik, M. G. Knepley, D. A. May, L. C. McInnes, R. T. Mills, T. Munson, K. Rupp, P. Sanan, B. F. Smith, S. Zampini, H. Zhang, and H. Zhang. PETSc users manual. Technical Report ANL-95/11 - Revision 3.13, Argonne National Laboratory, 2020. URL `https://www.mcs.anl.gov/petsc`.

[S7] Y. Bazilevs, V. Calo, J. Cottrell, T. Hughes, A. Reali, and G. Scovazzi. Variational multiscale residual-based turbulence modeling for large eddy simulation of incompressible flows. *Computer Methods in Applied Mechanics and Engineering*, 197(1-4):173–201, 2007. doi: 10.1016/j.cma.2007.07.016.

[S8] Y. Bazilevs, K. Takizawa, and T. Tezduyar. *Computational Fluid-Structure Interaction: Methods and Applications*. John Wiley and Sons, 2013. ISBN 9780470978771. doi: 10.1002/9781118483565.

[S9] M. Braack and P. B. Mucha. Directional do-nothing condition for the navier-stokes equations. *Journal of Computational Mathematics*, 32(5):507–521, June 2014. URL `https://doi.org/10.4208/jcm.1405-m4347`.

[S10] S. Brenner and R. Scott. *The mathematical theory of finite element methods*, volume 15. Springer Science & Business Media, 2007.

[S11] G. Carbou and P. Fabrie. Boundary layer for a penalization method for viscous incompressible flow. *Adv. Differential Equations*, 8(12):1453–1480, 2003.

[S12] C. Chnafa, S. Mendez, and F. Nicoud. Image-based large-eddy simulation in a realistic left heart. *Computers & Fluids*, 94:173–187, May 2014. URL `https://doi.org/10.1016/j.compfluid.2014.01.030`.

[S13] T. Engels, D. Kolomenskiy, K. Schneider, and J. Sesterhenn. FluSI: A novel parallel simulation tool for flapping insect flight using a fourier method with volume penalization. *SIAM Journal on Scientific Computing*, 38(5):S3–S24, Jan. 2016. URL `https://doi.org/10.1137/15m1026006`.

[S14] M. Esmaily-Moghadam, T.-Y. Hsia, and A. L. Marsden. A non-discrete method for computation of residence time in fluid mechanics simulations. *Physics of Fluids*, 25(11):110802, nov 2013. URL `https://doi.org/10.1063/1.4819142`.

[S15] L. Formaggia and F. Nobile. Stability analysis of second-order time accurate schemes for ALE–FEM. *Computer Methods in Applied Mechanics and Engineering*, 193(39-41):4097–4116, Oct. 2004. URL `https://doi.org/10.1016/j.cma.2003.09.028`.

[S16] J. Fouchet-Incaux. Artificial boundaries and formulations for the incompressible navier–stokes equations: applications to air and blood flows. *SeMA Journal*, 64(1):1–40, Jan. 2014. URL https://doi.org/10.1007/s40324-014-0012-y.

[S17] J. Fuchsberger, P. Aigner, S. Niederer, G. Plank, H. Schima, G. Haase, and E. Karabelas. On the incorporation of obstacles in a fluid flow problem using a navier–stokes–brinkman penalization approach. *Journal of Computational Science*, 57:101506, Jan. 2022. URL https://doi.org/10.1016/j.jocs.2021.101506.

[S18] E. Haines. Point in polygon strategies. In *Graphics Gems*, pages 24–46. Elsevier, 1994. URL https://doi.org/10.1016/b978-0-12-336156-1.50013-6.

[S19] V. E. Henson and U. M. Yang. BoomerAMG: A parallel algebraic multigrid solver and preconditioner. *Applied Numerical Mathematics*, 41(1):155–177, Apr. 2002. URL https://doi.org/10.1016/s0168-9274(01)00115-5.

[S20] C. Hirt, A. Amsden, and J. Cook. An arbitrary lagrangian-eulerian computing method for all flow speeds. *Journal of Computational Physics*, 14(3):227–253, Mar. 1974. URL https://doi.org/10.1016/0021-9991(74)90051-5.

[S21] S. Hodis, S. Uthamaraj, A. L. Smith, K. D. Dennis, D. F. Kallmes, and D. Dragomir-Daescu. Grid convergence errors in hemodynamic solution of patient-specific cerebral aneurysms. *Journal of Biomechanics*, 45(16):2907 – 2913, 2012. ISSN 0021-9290. doi: https://doi.org/10.1016/j.jbiomech.2012.07.030.

[S22] T. J. Hughes, W. K. Liu, and T. K. Zimmermann. Lagrangian-eulerian finite element formulation for incompressible viscous flows. *Computer Methods in Applied Mechanics and Engineering*, 29(3): 329–349, Dec. 1981. URL https://doi.org/10.1016/0045-7825(81)90049-9.

[S23] K. E. Jansen, C. H. Whiting, and G. M. Hulbert. A generalized-α method for integrating the filtered navier–stokes equations with a stabilized finite element method. *Computer Methods in Applied Mechanics and Engineering*, 190(3-4):305–319, Oct. 2000. URL https://doi.org/10.1016/s0045-7825(00)00203-6.

[S24] Y. Jin, S. Chai, J. Duffy, C. Chin, and N. Bose. Urans predictions of wave induced loads and motions on ships in regular head and oblique waves at zero forward speed. *Journal of Fluids and Structures*, 74:178 – 204, 2017. ISSN 0889-9746. doi: https://doi.org/10.1016/j.jfluidstructs.2017.07.009.

[S25] V. John and E. Schmeyer. Finite element methods for time-dependent convection–diffusion–reaction equations with small diffusion. *Computer Methods in Applied Mechanics and Engineering*, 198(3-4):475–494, Dec. 2008. URL https://doi.org/10.1016/j.cma.2008.08.016.

[S26] E. Karabelas, M. A. F. Gsell, C. M. Augustin, L. Marx, A. Neic, A. J. Prassl, L. Goubergrits, T. Kuehne, and G. Plank. Towards a computational framework for modeling the impact of aortic coarctations upon left ventricular load. *Frontiers in Physiology*, 9, May 2018. URL https://doi.org/10.3389/fphys.2018.00538.

[S27] E. Karabelas, G. Haase, G. Plank, and C. M. Augustin. Versatile stabilized finite element formulations for nearly and fully incompressible solid mechanics. *Computational Mechanics*, 65(1): 193–215, Sept. 2019. URL https://doi.org/10.1007/s00466-019-01760-w.

[S28] V. Kolář. Vortex identification: New requirements and limitations. *International Journal of Heat and Fluid Flow*, 28(4):638–652, Aug. 2007. URL https://doi.org/10.1016/j.ijheatfluidflow.2007.03.004.

[S29] D. Kuzmin. Explicit and implicit FEM-FCT algorithms with flux linearization. *Journal of Computational Physics*, 228(7):2517–2534, Apr. 2009. URL https://doi.org/10.1016/j.jcp.2008.12.011.

[S30] D. Kuzmin and M. Möller. Algebraic flux correction i. scalar conservation laws. In *Flux-Corrected Transport*, pages 155–206. Springer-Verlag, 2008. URL https://doi.org/10.1007/3-540-27206-2_6.

[S31] C. C. Long, M. Esmaily-Moghadam, A. L. Marsden, and Y. Bazilevs. Computation of residence time in the simulation of pulsatile ventricular assist devices. *Computational Mechanics*, 54(4): 911–919, sep 2013. URL https://doi.org/10.1007/s00466-013-0931-y.

[S32] P. W. Longest and S. Vinchurkar. Effects of mesh style and grid convergence on particle deposition in bifurcating airway models with comparisons to experimental data. *Medical Engineering & Physics*, 29(3):350 – 366, 2007. ISSN 1350-4533. doi: https://doi.org/10.1016/j.medengphy.2006.05.012.

[S33] L. Marx, M. A. Gsell, A. Rund, F. Caforio, A. J. Prassl, G. Toth-Gayor, T. Kuehne, C. M. Augustin, and G. Plank. Personalization of electro-mechanical models of the pressure-overloaded left ventricle: Fitting of Windkessel-type afterload models: Fitting of Windkessel afterload models. *Philos. Trans. R. Soc. A Math. Phys. Eng. Sci.*, 378(2173), 2020. ISSN 1364503X. URL https://doi.org/10.1098/rsta.2019.0342.

[S34] M. E. Moghadam, , Y. Bazilevs, T.-Y. Hsia, I. E. Vignon-Clementel, and A. L. Marsden. A comparison of outlet boundary treatments for prevention of backflow divergence with relevance to blood flow simulations. *Computational Mechanics*, 48(3):277–291, Apr. 2011. URL https://doi.org/10.1007/s00466-011-0599-0.

[S35] T. Möller and B. Trumbore. Fast, minimum storage ray-triangle intersection. *Journal of Graphics Tools*, 2(1):21–28, Jan. 1997. URL https://doi.org/10.1080/10867651.1997.10487468.

[S36] A. Neic, M. A. Gsell, E. Karabelas, A. J. Prassl, and G. Plank. Automating image-based mesh generation and manipulation tasks in cardiac modeling workflows using meshtool. *SoftwareX*, 11:100454, Jan. 2020. URL https://doi.org/10.1016/j.softx.2020.100454.

[S37] L. H. Pauli. *Stabilized Finite Element Methods for ComputationalDesign of Blood-Handling Devices.* PhD thesis, 6 2016.

[S38] S. B. Pope. Ten questions concerning the large-eddy simulation of turbulent flows. *New Journal of Physics*, 6:35–35, Mar. 2004. URL https://doi.org/10.1088/1367-2630/6/1/035.

[S39] N. Scuro, E. Angelo, G. Angelo, and D. Andrade. A cfd analysis of the flow dynamics of a directly-operated safety relief valve. *Nuclear Engineering and Design*, 328:321 – 332, 2018. ISSN 0029-5493. doi: https://doi.org/10.1016/j.nucengdes.2018.01.024.

[S40] O. Steinbach. *Numerical approximation methods for elliptic boundary value problems: finite and boundary elements.* Springer Science & Business Media, 2007.

[S41] P. L. Tallec and J. Mouro. Fluid structure interaction with large structural displacements. *Computer Methods in Applied Mechanics and Engineering*, 190(24-25):3039–3067, Mar. 2001. URL https://doi.org/10.1016/s0045-7825(00)00381-9.

[S42] E. Vigmond, R. W. dos Santos, A. Prassl, M. Deo, and G. Plank. Solvers for the cardiac bidomain equations. *Progress in Biophysics and Molecular Biology*, 96(1-3):3–18, Jan. 2008. URL https://doi.org/10.1016/j.pbiomolbio.2007.07.012.

[S43] E. J. Vigmond, M. Hughes, G. Plank, and L. Leon. Computational tools for modeling electrical activity in cardiac tissue. *Journal of Electrocardiology*, 36:69–74, Dec. 2003. URL `https://doi.org/10.1016/j.jelectrocard.2003.09.017`.

[S44] S. T. Zalesak. Fully multidimensional flux-corrected transport algorithms for fluids. *Journal of Computational Physics*, 31(3):335–362, June 1979. URL `https://doi.org/10.1016/0021-9991(79)90051-2`.
